# The cyclooxygenase-1/mPGES-1/endothelial prostaglandin EP4 receptor pathway constrains myocardial ischemia-reperfusion injury

**DOI:** 10.1038/s41467-019-09492-4

**Published:** 2019-04-23

**Authors:** Liyuan Zhu, Chuansheng Xu, Xingyu Huo, Huifeng Hao, Qing Wan, Hong Chen, Xu Zhang, Richard M. Breyer, Yu Huang, Xuetao Cao, De-Pei Liu, Garret A. FitzGerald, Miao Wang

**Affiliations:** 1 State Key Laboratory of Cardiovascular Disease, Fuwai Hospital, National Center for Cardiovascular Diseases, Chinese Academy of Medical Sciences and Peking Union Medical College, Beijing, 100037 China; 2 Tianjin Key Laboratory of Metabolic Diseases and Department of Physiology, Tianjin Medical University, Tianjin, 300070 China; 3 Division of Nephrology, Department of Medicine, Vanderbilt University Medical Center, Nashville, TN 37212 USA; 4 Institute of Vascular Medicine and Li Ka Shing Institute of Health Sciences, The Chinese University of Hong Kong, Hong Kong SAR, China; 5 Institute of Basic Medical Sciences, Chinese Academy of Medical Sciences and Peking Union Medical College, Beijing, 100005 China; 6 Institute for Translational Medicine and Therapeutics, Perelman School of Medicine, Department of Systems Pharmacology and Translational Therapeutics, University of Pennsylvania, Philadelphia, PA 19104 USA; 7 Clinical Pharmacology Center, Fuwai Hospital, National Center for Cardiovascular Diseases, Chinese Academy of Medical Sciences and Peking Union Medical College, Beijing, 100037 China

**Keywords:** Pharmacology, Target identification, Cardiovascular biology, Circulation

## Abstract

The use of nonsteroidal anti-inflammatory drugs that inhibit cyclooxygenase (COX)-1 and COX-2, increases heart failure risk. It is unknown whether microsomal (m) prostaglandin (PG) E synthase (S)-1, a target downstream of COX, regulates myocardial (M) ischemia/reperfusion (I/R) injury, a key determinant of heart failure. Here we report that COX-1 and mPGES-1 mediate production of substantial amounts of PGE_2_ and confer cardiac protection in MI/R. Deletion of *mPges-1* impairs cardiac microvascular perfusion and increases inflammatory cell infiltration in mouse MI/R. Consistently, *mPges-1* deletion depresses the arteriolar dilatory response to I/R in vivo and to acetylcholine ex vivo, and enhances leukocyte-endothelial cell interaction, which is mediated via PGE receptor-4 (EP4). Furthermore, endothelium-restricted *Ep4* deletion impairs microcirculation, and exacerbates MI/R injury, irrespective of EP4 agonism. Treatment with misoprostol, a clinically available PGE analogue, improves microcirculation and reduces MI/R injury. Thus, mPGES-1, a key microcirculation protector, constrains MI/R injury and this beneficial effect is partially mediated via endothelial EP4.

## Abstract (web summary)

The use of nonsteroidal anti-inflammatory drugs inhibiting COX-1/2 is associated with an increased risk of heart failure. Here the authors show that mPGES-1, a therapeutic target downstream of COX enzymes, protects from cardiac ischemia/reperfusion injury, limiting leukocyte-endothelial interactions and preserving microvascular perfusion partly via the endothelial EP4 receptor.

## Introduction

Nonsteroidal anti-inflammatory drugs (NSAIDs) ameliorate pain and fever by inhibiting cyclooxygenase (COX) activity, thus suppressing biosynthesis of prostanoids. COX (which exists mainly in two isoforms, COX-1 and COX-2) catalyzes the formation of prostaglandin (PG) H_2_, the common substrate for downstream isomerases to synthesize individual prostanoids, including PGE_2_ and PGI_2_. Use of NSAIDs is associated with an increased risk of heart failure^1^. The role of COX pathway in myocardial (M) ischemia/reperfusion (I/R) injury, a key determinant of the subsequent development of heart failure^2^, remains unclear. MI/R also constitutes a therapeutic target under intensive investigation^2–4^.

Microsomal (m) PGE synthase (S)-1, the dominant synthetic enzyme for PGE_2_ production in vivo^5^, is being considered as a new therapeutic target downstream of COX^6–8^. In contrast to COX-2 inhibitors, deletion of *mPges-1* augments PGI_2_ production,  restraining thrombogenesis and atherogenesis, and modulating the response to vascular injury^5,9–12^. In isolated mouse hearts, both COX-1 and COX-2 mediate recovery of left ventricular developed pressure after ischemia^13^. However, in mice, COX-2 inhibition does not modify infarct size post MI/R^14^, and the role of COX-1 remains undetermined in vivo. Both PGI_2_ and PGE_2_ may be formed by COXs in MI/R^13,15^. PGI_2_ restrains MI/R injury via its receptor (IP)^16^. In a mouse model of sustained (4 weeks) myocardial infarction, i.e., without reperfusion after coronary occlusion, global *mPges-1* deletion^17^, or its deletion in bone marrow-derived leukocytes^18^, impairs chronic cardiac remodeling. However, *mPges-1* deletion does not worsen cardiac ischemic injury after 24 h coronary occlusion^19^. It remains unknown whether mPGES-1 regulates myocardial I/R injury, a clinically relevant pathologic process in patients with myocardial infarction undergoing reperfusion therapy^2^. PGE_2_ has four PGE receptors (EP1-4) mediating diverse biological functions^20^. Global deletion of *Ep4* increases infarct size post MI/R^21^, whereas cardiomyocyte-specific *Ep4* deletion reduces cardiac hypertrophy without changing infarct size in a model of sustained MI^22^, suggesting the potential involvement of cells other than cardiomyocytes in mediating the impact of global *Ep4* KO.

Here we report that mPGES-1 protects against acute MI/R injury in mice and this is attributed to its critical role in preserving microcirculation and limiting inflammation in I/R. The cardioprotective effect of mPGES-1 is partially mediated through PGE_2_ action on the endothelial EP4 receptor. Inhibition of COX-1/mPGES-1-derived PGE_2_ contributes to a risk of myocardial injury in the setting of MI/R.

## Results

### COX-1 protects against MI/R injury in mice

**Fig. 1:**
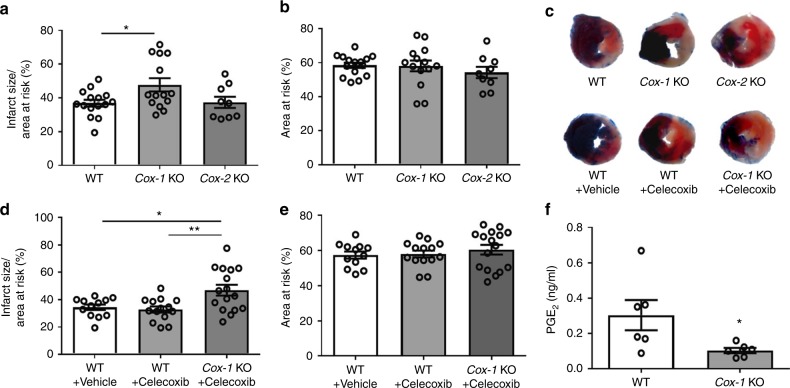
Impact of *Cox-1/2* deletion on MI/R injury. Infarct size (IS) (**a**) and area at risk (AAR) (**b**) were quantified 24 h after I/R injury, as detailed in the Methods. Representative photographs showing TTC stained transverse sections of Evans blue perfused hearts were shown for each study group (**c**). In a separate study, mice were orally treated with 100 mg/kg celecoxib or vehicle before and 20 h after surgery. Representative TTC staining of the heart sections was shown for each group (**c**), and their infarct size (**d**) and AAR (**e**) were quantified. Biosynthesis of PGE_2_ (**f**) was measured by HPLC-MS-MS as detailed in the Methods. One-way ANOVA with Tukey’s multiple comparison test (**a**, **b**, *n* = 15, 14, 9; **d**, **e**, *n* = 12, 14, 16. Individual *n* numbers in each panel are shown in same order as their corresponding groups from left to right, and this arrangement is applied to other relevant figures in the text.). Unpaired Student’s *t* test (**f**, *n* = 6). Statistical significance is demarked as *P < 0.05, **P < 0.01, and ***P < 0.001, throughout the text. Error bar indicates SEM

COX is the rate-limiting enzyme upstream of mPGES-1. We set out to examine the relative importance of COX-1 and COX-2 in MI/R in vivo. *Cox-1* KO and *Cox-2* KO mice and their littermates were subjected to 30-min ischemia by ligating the left anterior descending coronary artery, followed by 24-h reperfusion. The same MI/R protocol was used throughout this study. Deletion of *Cox-1*, but not *Cox-2* significantly increased the percentage of infarct area in the area at risk (AAR), with AAR similar across the groups (Fig. 1a–c). Treatment with celecoxib, a COX-2 selective inhibitor, at 100 mg/kg, a dose previously shown to accelerate experimentally induced thrombosis^5^, did not modify infarct size, whereas *Cox-1* deletion increased infarct size after MI/R injury (Fig. 1c–e), confirming a protective role of COX-1 against MI/R injury. *Cox-1* deletion suppressed formation of PGE_2_ (Fig. 1f)_,_ a potential mediator of the cardioprotection of COX-1. MI/R increased urinary excretion of PGE_2_ metabolites, which was mainly produced by COX-1, not COX-2 (Supplementary Fig. 1A, B). Consistently, COX-1 is the more abundant isoform expressed in the heart (Supplementary Fig. 1C), also as previously reported^13,23^.

### mPGES-1 protects against MI/R injury

**Fig. 2:**
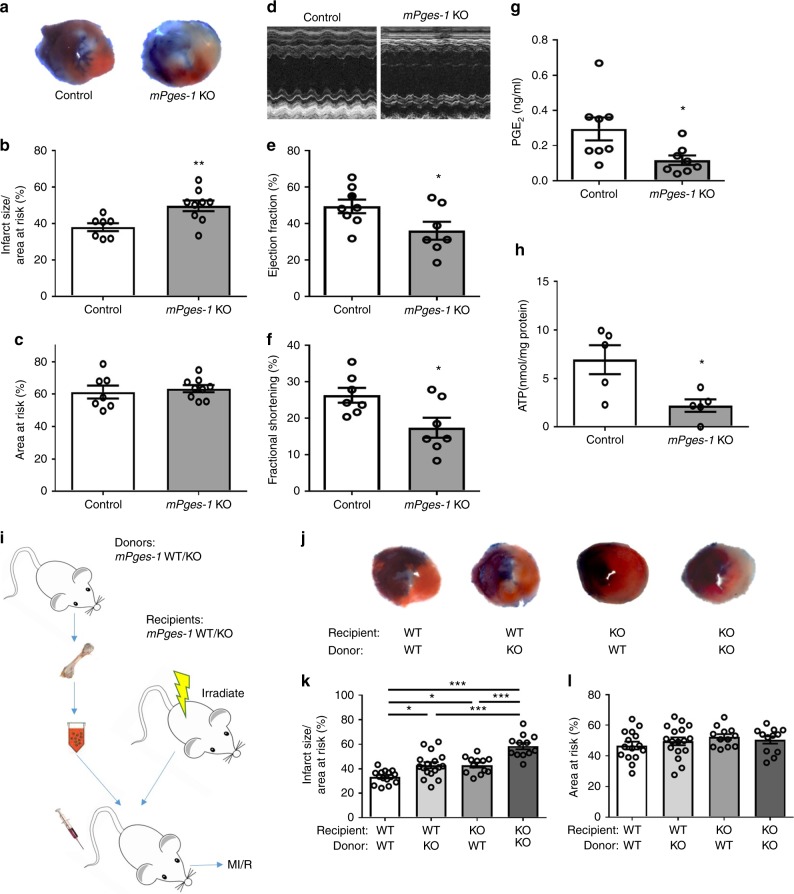
Impact of *mPges-1* deletion on MI/R injury. Mice were subjected to MI/R injury as detailed in the Methods. **a** Representative photographs of TTC stained sections from Evans blue perfused hearts. Infarct size (**b**) and AAR (**c**) were quantified for *mPges-1* KO and control mice. Representative echocardiograph (**d**), ejection fraction (EF) (**e**), and fractional shortening (FS) (**f**) were shown. PGE_2_ levels were compared (**g**). Cardiac levels of ATP post MI/R were measured (**h**). Unpaired Student’s *t* test (**b**, **c**, *n* = 7, 9; **e**, *n* = 8, 7; **f**, *n* = 7, 7; **g**, *n* = 8, 8; **h**, *n* = 5). Bone marrow cells were transplanted reciprocally between *mPges-1* WT and KO mice (**i**). The resulting chimeric mice underwent MI/R injury. Representative TTC staining was shown for each group (**j**). Infarct size (**k**) and AAR (**l**) were analyzed with use of one-way ANOVA and Tukey’s multiple comparison test (*n* = 15, 17, 12, 12). Error bar indicates SEM

We next focused on the role of mPGES-1 in MI/R injury. Deletion of *mPges-1* increased infarct size (Fig. 2a–c) and reduced fractional shortening and ejection fraction (Fig. 2d–f). Naive *mPges-1* KO mice showed no difference in ultrasonographic parameters compared with controls (Supplementary Fig. 2A–D). Again, deletion of *mPges-1* suppressed the level of PGE_2_ (Fig. 2g), suggesting an integrative cardioprotective mechanism of COX-1 and mPGES-1 in the setting of MI/R. Measurement of urinary metabolites of PGE_2_ and PGI_2_ indicated mPGES-1 as a major source of PGE_2_ and a shunting toward biosynthesis of PGI_2_ in the face of PGE_2_ suppression, during MI/R (Supplementary Fig. 2E, F). The role of PGE_2_ suppression in mediating the deleterious effect of *mPges-1* deletion on MI/R injury was further confirmed by treating the animals with misoprostol, a PGE analog, which abolished the exaggerated infarct size due to *mPges-1* deletion (Supplementary Fig. 3). Tissue levels of ATP in the area of myocardium at risk after I/R were reduced in *mPges-1* KO mice (Fig. 2h) while the ratio of ADP to ATP was increased (Supplementary Fig. 4A, B). Plasma levels of IL22 were not altered by *mPges-1* deletion in MI/R (Supplementary Fig. 4C).

Reciprocal bone-marrow transplantation was conducted between *mPges-1* WT and KO mice (Fig. 2i). Specific deletion of *mPges-1* in bone marrow-derived cells (BMCs) or in non-BMCs increased infarct size post MI/R injury, and cardiac injury was further exacerbated by the loss of *mPges-1* in both compartments (Fig. 2j–l). Impaired ejection fraction and fractional shortening were observed in the chimeric mice with *mPges-1* KO in bone marrow or in both compartments, compared with WT to WT chimeric mice (Supplementary Fig. 5A–C). Left ventricular contractile function correlated with infarct size (Supplementary Fig. 5D). Hence, both BMC and non-BMC mediated the protective role of mPGES-1 in restraining infarct size in MI/R injury.

### *mPges-1* deletion impairs microvascular perfusion in MI/R

**Fig. 3:**
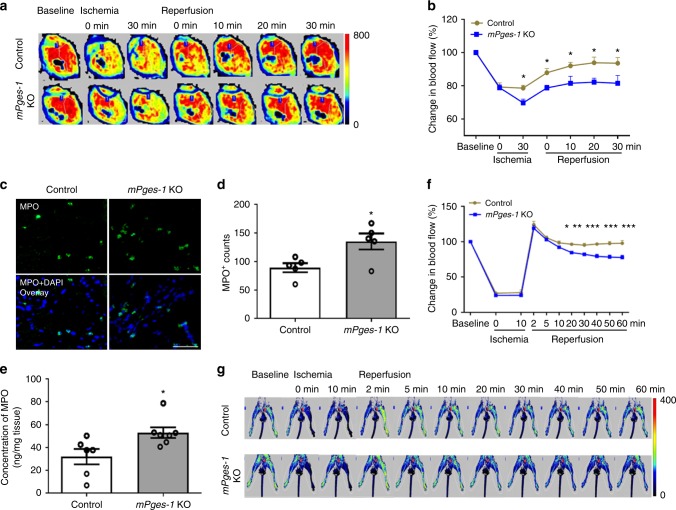
Essential role of mPGES-1 in preserving microcirculation during I/R. Cardiac microvascular perfusion (percentage change relative to baseline) was recorded at indicated time points before and after release of coronary ligation in *mPges-1* WT and KO mice, as detailed in the Methods. Representative perfusion images at each time point were shown (**a**). Relative change in blood flow was analyzed (**b**). Immunofluorescent staining of myeloperoxidase (MPO, in green) was carried out on the heart sections, and representative photos were shown (**c**). MPO-positive cells (**d**) and tissue levels of MPO (**e**) in the ischemic area were quantified. Distal microvascular perfusion was also measured during hindlimb I/R. Microvascular flow relative to baseline was recorded at indicated time points before and after release of the artery ligation (**f**), with representative perfusion images at each time point shown (**g**). At the baseline before ischemia, no difference in cardiac perfusion (514 ± 13 vs 526 ± 18 PU, *n* = 17, 14, unpaired Student’s *t* test) or in hindlimb perfusion (86 ± 6 vs 90 ± 4 PU, *n* = 5, unpaired Student’s *t* test) was observed between control and *mPges-1* KO. Two-way ANOVA with Bonferroni’s test (**b**, *n* = 12, 11; **f**, *n* = 5). Unpaired Student’s *t* test (**d**, *n* = 5; **e**, *n* = 6, 7). Scale bar is 50 μm. Error bar indicates SEM

Microvascular dysfunction is one key determinant of MI/R injury^24–26^. We used laser Doppler flowmetry to record cardiac microvascular perfusion before and after release of coronary artery ligation. Deletion of *mPges-1* impaired cardiac perfusion following restoration of coronary blood flow (Fig. 3a, b), without affecting baseline cardiac perfusion. The injured heart tissue of *mPges-1* KO mice showed an increased number of myeloperoxidase (MPO)-positive cells analyzed by immunofluorescent staining (Fig. 3c, d), which was also confirmed by flow-cytometry analysis (Supplementary Fig. 6A, B; the gating strategy for neutrophils was shown in Supplementary Fig. 6C). Heart MPO levels were increased by MI/R (Supplementary Fig. 6D) and *mPges-1* deletion further increased MPO levels (Fig. 3e). Enhanced vascular permeability was also observed in the injured *mPges-1* KO hearts (Supplementary Fig. 6E). We utilized another mouse model, in which I/R was induced by ligating the femoral artery and tissue blood flow was monitored in the hindlimb. Deletion of *mPges-1* suppressed the tissue perfusion response relative to control (Fig. 3f, g). Taken together, these results suggest that impaired microcirculatory perfusion may underlie the enhanced MI/R injury in *mPges-1* KO mice (Fig. 2). We next examined whether mPGES-1 regulates vasoreactivity and leukocyte–endothelial cell interactions, both of which contribute to microcirculatory function in I/R^25^.

### mPGES-1 and EP4 mediate arteriolar dilation

**Fig. 4:**
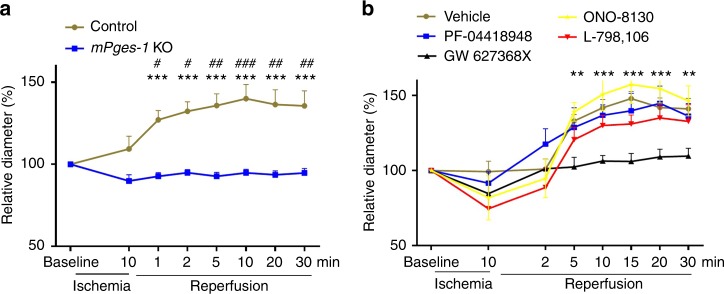
*mPges-1* deletion and PGE receptor antagonism in arteriolar dilation in I/R. Arterioles of 15–25 μm in diameter were randomly selected in the extremity of hindlimb. Vascular response (percentage change of diameter over baseline) to I/R was assessed for mice deficient in *mPges-1* (**a**). The vessel diameter was not different between control (*n* = 9) and *mPges-1-1 KO* (*n* = 7) mice at baseline (21.34 ± 0.72 vs 20.38 ± 1.40 μm, *p* = 0.54, unpaired Student’s *t* test). **b** The vascular response was also assessed for WT mice i.v. treated with vehicle (*n* = 6), or an antagonist EP1 (ONO-8130, Ki = 1.9 nM, *n* = 5), EP2 (PF-04418948, IC50 = 16 nM, *n* = 5), EP3 (L-798106, Ki = 0.3 nM, *n* = 7) or EP4 (GW627368X, Ki = 100 nM, *n* = 7), with each drug at 5 mg/kg. There was no difference in baseline vessel diameter between groups. Two-way ANOVA with Bonferroni’s test was used. * demarks statistical comparison between control and *mPges-1* KO, or between GW 627368X and vehicle. # shows comparison to baseline. Error bar indicates SEM

Arteriolar size was measured during hindlimb I/R in vivo. Reperfusion induced arteriolar dilatation in wild-type mice, however, this response was absent in *mPges-1* deficient mice (Fig. 4a). Treatment with GW627368X (an EP4 antagonist), but not antagonists of EP1, EP2 or EP3, inhibited the vascular dilatory response (Fig. 4b). We further characterized isometric vessel tension ex vivo. *mPges-1* deletion impaired endothelium-dependent relaxation in response to acetylcholine in resistance arteries, but not in aortas (Supplementary Fig. 7).

### mPGES-1/PGE_2_/EP4 axis limits leukocyte-EC interactions

**Fig. 5:**
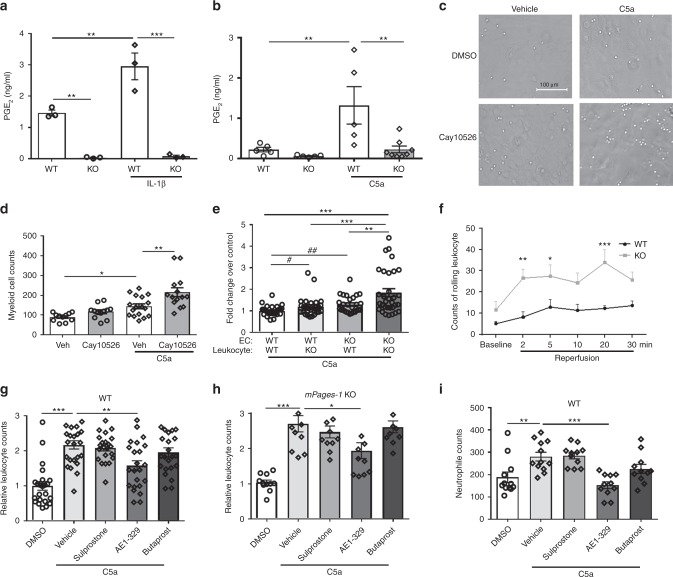
mPGES-1 derived PGE_2_ and EP4 activation limit leukocyte-EC interactions. PGE_2_ levels were measured for culture supernatant of mouse (*mPges-1* WT/KO) lung endothelial cells (ECs) (**a**) and peritoneal myeloid cells (**b**). Cells were treated with 10 ng/ml IL-1β for 12 h (**a**) or with 100 nM C5a for 30 min (**b**). Effect of mPGES-1 inhibition on cell adhesion was analyzed. WT ECs were treated with 10 μM Cay10526 for 12 h before peritoneal myeloid cells were applied, and cell adhesion was quantified in absence or presence of 100 nM C5a. Representative photos of cell adhesion for each treatment condition (**c**) and the quantitative results (**d**) were shown. Adhesion of blood leukocytes to ECs was determined in vitro in presence of 100 nM C5a (**e**). The fold change of adherent cells over the number for WT leukocyte to WT EC was presented, with cell genotype labeled below each column. Leukocyte–endothelial cell interactions were videotaped by intravital microscopy during hindlimb I/R, and rolling cells were counted prior to and after femoral artery ligation and reperfusion at indicated time points (**f**), as detailed in the Methods. Effects of agonists for EP1/3 (Sulprostone), EP2 (Butaprost), and EP4 (AE1-329), each at 1 μM, on in vitro blood leukocyte adhesion to WT (**g**) or *mPges-1* KO (**h**) EC monolayer were examined in presence of 100 nM C5a. Adhesion of purified blood neutrophils was similarly examined (**i**). One-way ANOVA with Tukey’s multiple comparison tests (**a**, *n* = 3; **e**, *n* = 29, 28, 27, 32; **g**, *n* = 23, 24, 22, 23, 23; **h**, *n* = 11, 11, 11, 11, 10; **i**, *n* = 12, 12, 11, 11, 11) or Bonferroni’s multiple comparison tests (**b**, *n* = 5, 5, 5, 8; **d**, *n* = 11, 12, 17, 14). Two-way ANOVA with Bonferroni’s tests (**f**, *n* = 6) and unpaired Student’s *t* test (**e**, *n* = 29, 28, 27, 32, ^#^*p* = 0.045; ^##^*p* = 0.0011). Error bar indicates SEM

Mouse lung ECs and peritoneal myeloid cells produced PGE_2_, mainly derived from mPGES-1, in response to IL-1β and complement component 5a (C5a), respectively (Fig. 5a, b). The myeloid cells were incubated over a monolayer of ECs and adherent cells were quantified. Treatment of ECs with 10 μM Cay10526 (an inhibitor of PGE_2_ production through the selective modulation of mPGES-1 expression, IC_50_ = 1.8 µM^27^) increased cell adhesion, particularly in the presence of C5a (Fig. 5c, d). C5a-stimulated blood leukocyte adhesion was also increased by *mPges-1* deficiency in ECs, as well as in leukocytes (Fig. 5e). Furthermore, I/R of mouse hindlimb induced leukocyte rolling, and such interactions were also increased by *mPges-1* deletion (Fig. 5f). Thus, mPGES-1-derived PGE_2_ limits leukocyte–endothelial cell interactions in the setting of I/R.

To determine which EP receptor(s) mediate these EC–leukocyte interactions, cells were treated with agonists^28,29^ for EP1/3 (sulprostone, EC_50_ = 0.42 nM for EP3, also a weak agonist for EP1), EP2 (butaprost, EC_50_ = 32 nM) or EP4 (AE1-329, EC_50_ = 3.1 nM), each at 1 μM. The EP4 agonist attenuated leukocyte adhesion to ECs from both WT (Fig. 5g) and *mPges-1* KO mice (Fig. 5h). Similar effects were observed when isolated blood neutrophils were used (Fig. 5i).

Freshly prepared human neutrophils were assessed for adhesion to human microvascular ECs in vitro. Treatment of ECs with Cay10256 increased C5a-induced neutrophil adhesion to the EC monolayer, and activation of EP4 potently inhibited this response, in the absence or presence of Cay10256 (Supplementary Fig. 8).

### EC EP4 protects microcirculation and the heart in MI/R

**Fig. 6:**
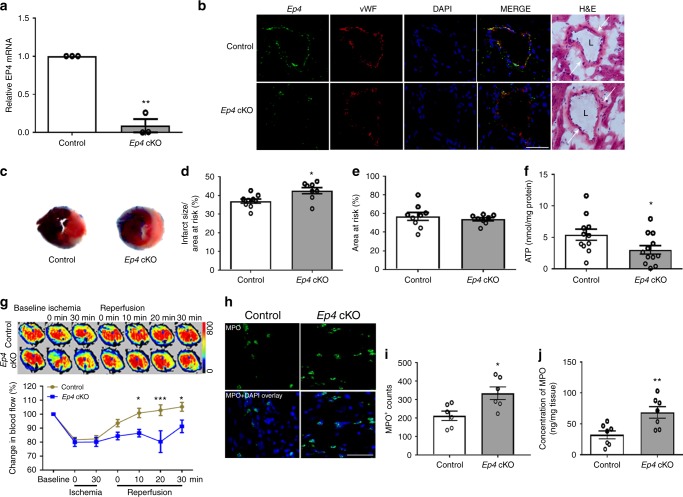
EC *Ep4* deletion impairs microcirculation and increases infarct size in MI/R. Expression of *Ep4* was examined by RT-PCR in the mouse lung ECs isolated from tamoxifen treated *Ep4* conditional KO mice (cKO) and littermate controls (**a**). Hearts were isolated 24 h after MI/R, and tissue sections were stained for EP4 (green), vWF (an EC marker, red) and nuclei (DAPI, blue). Representative result from three studies was shown (**b**). Representative TTC stained sections of the Evans blue perfused hearts were shown (**c**). Infarct size (**d**) and AAR (**e**) were quantified. Levels of ATP in the injured myocardium 24 h post IR were measured (**f**). Cardiac blood perfusion during I/R was measured by laser Doppler flow, and representative images were shown above the flow plot (**g**). Tissue expression of MPO was stained (**h**) and the number of MPO-positive cells was analyzed (**i**). Heart tissue levels of MPO in the ischemic area were quantified (**j**). Unpaired Student’s *t* test (**a**, *n* = 3; **d**, **e**, *n* = 9, 8; **f**, *n* = 11, 12; **i**, *n* = 6; **j**, *n* = 7). Two-way ANOVA with Turkey’s tests (**g**, *n* = 15, 7). Scale bar is 50 μm. Anatomy of vascular structure is labeled in **b**: arrows denote vessel wall, and ‘L’ denotes lumen. Error bar indicates SEM

Given that EP4 mediated flow-induced arteriolar dilation (Fig. 4) and leukocyte-EC interactions (Fig. 5), we next examined the role of endothelial EP4 in MI/R using mice with postnatal deletion of endothelial *Ep4* (EC cKO). Expression of *Ep4* mRNA was reduced in ECs isolated from EC cKO mice (Fig. 6a). In hearts after MI/R, EP4 was co-stained with vWF (an EC marker) in control mice, and its expression was reduced in the cKOs (Fig. 6b). Further staining showed low cardiac expression of EP1 and EP2, discrete expression of EP3 and high expression of EP4 in ECs (Supplementary Fig. 9A). Loss of endothelial *Ep4* increased infarct size after MI/R (Fig. 6c–e). Levels of ATP (Fig. 6f) in the injured myocardium were reduced in *Ep4* cKO mice along with increased levels of ADP and the ratio of ADP to ATP (Supplementary Fig. 6B, C). This coincided with impaired cardiac microvascular perfusion following MI/R (Fig. 6g). More MPO-positive cells were stained in heart sections of the cKO mice (Fig. 6h, i) and cardiac levels of MPO were also increased (Fig. 6j).

We further evaluated the effect of an EP4 agonist (Cay10580) and an EP4 antagonist (L161,982) on MI/R injury in *Ep4* EC cKO and littermate controls (Supplementary Fig. 10A–C). Treatment with Cay10580 reduced infarct size in both control mice and the cKO mice, however, the cKOs still showed increased injury compared with controls despite the EP4 agonism. EP4 blockade with L161,982 increased infarct size in control mice, to an extent similar to that observed in cKO mice. Similar proportional increase in infarct size was also observed in mice with postnatal global *Ep4* deletion (Supplementary Fig. 10D–F). Therefore, EP4 in the endothelium plays a critical, but not exclusive, role in mediating the cardioprotective effect of PGE_2_ in MI/R.

### Misoprostol protects the heart in MI/R

**Fig. 7:**
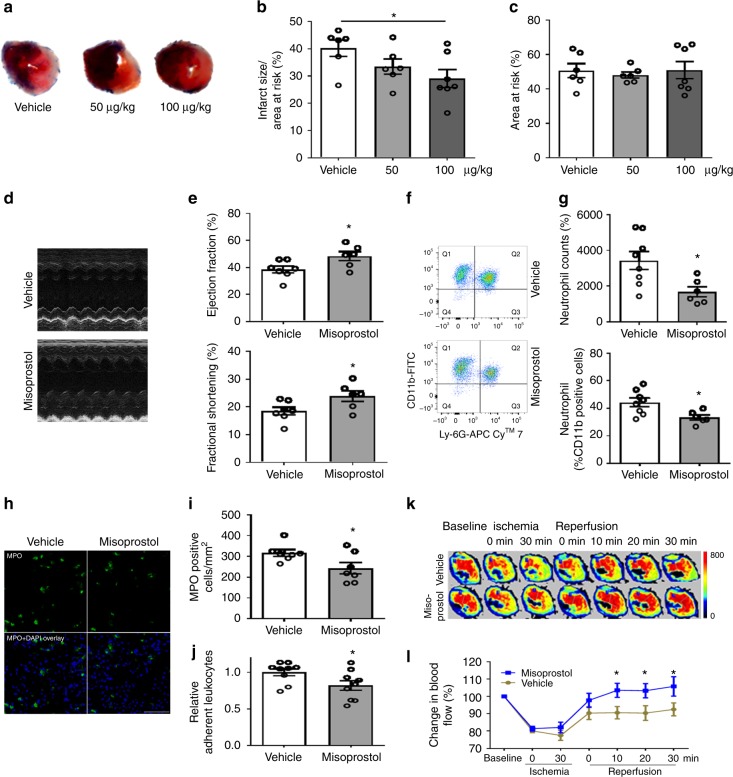
Misoprostol improves microcirculation and protects the heart in MI/R. WT mice were subjected to MI/R surgery. Vehicle, 50 or 100 μg/kg misoprostol was injected i.p. following LAD ligation before reperfusion, and additionally at 4, 8, 12 h of reperfusion. Representative photos of TTC stained Evans blue perfused hearts were shown (**a**). Infarct size (**b**) and AAR (**c**) were quantified. Ultrasonography was carried out at 24-h post MI/R (**d**). Ejection fraction (EF) and fractional shortening (FS) were quantified (**e**). Neutrophils (CD11b^+^Ly-6G^+^) in the ischemic heart tissue were detected by flow cytometry (**f**) and quantified as cell counts and percent of neutrophils in CD11b^+^ cells (**g**). Immunofluorescent staining of MPO was performed on the heart sections (**h**) and number of MPO-positive cells was quantified (**i**). Effect of misoprostol (10 μM) on leukocyte adhesion to ECs in vitro (**j**). In another set of mice, myocardial microcirculatory perfusion was determined during MI/R, and the representative perfusion images (**k**) and the statistical graph (**l**) were shown. One-way ANOVA with Dunn’s multiple comparison test (**b**, **c**, *n* = 6, 6, 7). Unpaired Student’s *t* test (**e**, *n* = 6, 7; **g**, *n* = 6, 8; **i**, *n* = 7; **j**, *n* = 9). Two-way ANOVA with Bonferroni’s test (**l**, *n* = 16, 15). Scale bar is 50 μm. Error bar indicates SEM

Misoprostol is a PGE analog clinically used to treat NSAIDs (including aspirin)-induced gastric ulcers. It inhibits formation of gastric lesions in rats (ED_50_ = 0.31 µg/kg). When administered following coronary artery ligation, misoprostol (100 µg/kg) reduced infarct size (Fig. 7a–c) and improved cardiac function after MI/R in mice (Fig. 7d, e). Flow-cytometry analysis of the injured heart revealed a reduction in infiltrated neutrophils (CD11b^+^Ly-6G^+^) (absolute counts: 3430 ± 502 vs 1681 ± 279; percentage of neutrophils in CD11b^+^ cells: 44.3 ± 3.1 vs 33.4 ± 1.9%) after MI/R injury (Fig. 7f, g). MPO-positive cells in the heart sections were also reduced by misoprostol treatment (Fig. 7h, i). Misoprostol inhibited leukocyte adhesion in vitro (Fig. 7j) and improved microcirculation following reperfusion (Fig. 7k, l and Supplementary Fig. 11). These observations indicate a key role of PGE_2_ signaling in preserving microcirculation, while raising the possibility that misoprostol might be repositioned to treat MI/R injury.

## Discussion

In this study, we reveal the cardioprotective effect of mPGES-1 in acute MI during cardiac reperfusion. This benefit is attributed to mPGES-1 protection of the microcirculation in I/R, which is mediated through the endothelial EP4 receptor. Timely and effective perfusion is critical to salvaging ischemic heart in acute MI, and deficiency in microvascular function is associated with clinical events^24,30,31^. Using a standard 30-min ischemia/24-h reperfusion protocol, this study was focused on acute reperfusion injury, a clinically relevant pathology that might be therapeutically targeted. An extended period after I/R would further increase the clinical relevance of this study. mPGES-1 is required for efficient reperfusion in MI/R.

The microcirculatory protection of mPGES-1 in I/R may utilize two mechanisms. First, mPGES-1 mediates resistance artery dilatation. COX inhibition-sensitive prostaglandins of unknown identity were reported to mediate arteriolar dilatation^32^. PGI_2_ is a potent vasodilator^28^ and regulates coronary blood flow in human and mouse^25,33^. PGE_2_ was reported either to dilate (EP2, EP4) or constrict (EP1, EP3) large blood vessels depending on vessel types and the receptor subtypes involved^34,35^. Inhibition of mPGES-1 in isolated large arteries reduces noradrenaline-induced constriction by increasing PGI_2_^36^. Here, we directly measured the diameter of resistance vessels in response to reperfusion. This pathophysiological condition-associated arteriolar dilatation was EP4-dependent, and was abolished by *mPges-1* deletion (Fig. 4). A functional study ex vivo provided further evidence that endothelium-dependent dilatation is likely mediated by mPGES-1 in resistance arteries, but not in conduit arteries (Supplementary Fig. 7). Second, mPGES-1-derived PGE_2_ and EP4 activation limited leukocyte-EC interactions (Fig. 5, Supplementary Fig. 8), adding an additional mechanism for the microcirculatory protection of mPGES-1 in I/R.

The microcirculatory perfusion detected by laser Doppler flow, reflects a combined signal from both ischemic area (due to LAD ligation) and nearby non-ischemic area (flow supplied by other branches of the coronary artery). Interestingly, reduced microcirculatory perfusion was also observed during the cardiac ischemia phase in *mPges-1* KOs (Fig. 3b). This likely reflects an mPGES-1 mediated microcirculatory adaption of the non-ischemic area to the increased metabolic demand of the ischemic area^26^, which itself indicates a critical role of mPGES-1 in the regulation of microcirculation. However, such pathophysiological compensation in the non-ischemic area might contribute little, if any, to the infarct size. This is because (1) mPGES-1exerts an essential and robust effect in microvascular dilation in the vessels directly affected by I/R (Fig. 4); (2) loss of *mPges-1* triggers a progressive defect in distal limb microcirculation following reperfusion, but without any effect during the ischemia phase (Fig. 3f); and (3) as shown previously, *mPges-1* deletion does not affect infarct size in mice undergoing acute ischemia only, i.e., without reperfusion^19^. Therefore, preservation of the microcirculation by mPGES-1 during the reperfusion (but not ischemic) phase substantially contributes to the protection against MI/R injury.

**Fig. 8:**
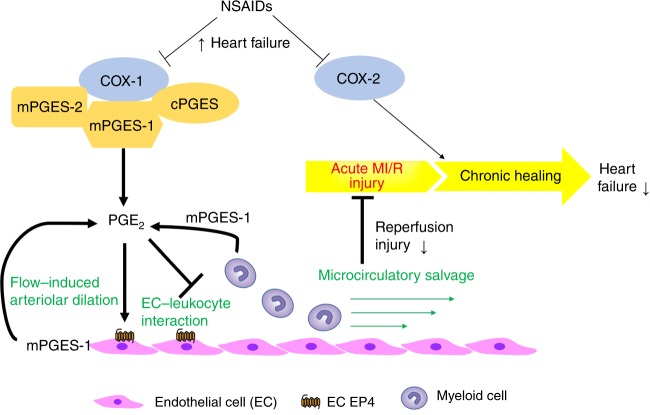
Schematic illustration of the role of mPGES-1 and EC EP4 in MI/R. Use of NSAIDs is associated with heart failure. In MI/R related heart failure, the COX pathway protects against development of heart failure through acute protection mainly via COX-1/mPGES-1, as shown in the present study (key components/steps highlighted in bold text), and through chronic healing mainly via COX-2, as shown in previous studies. COX-1, the constitutive COX isoform and mPGES-1 contribute substantially to PGE_2_ biosynthesis and limit acute MI/R injury. mPGES-1, the major source of PGE_2_ in endothelial cells and myeloid cells, plays a pivotal role in mediating flow-induced arteriolar dilation and in limiting EC–leukocyte interactions, under I/R. By doing so, mPGES-1 preserves the microcirculation and further constrains MI/R injury. These beneficial effects are mediated in part via endothelial EP4 receptors. → denotes ‘prostanoid production’ or ‘positive regulation’; ┤denotes ‘inhibition’; the thick lines highlight the key findings of this study. cPGES: cytosolic PGE synthase

The protective role of mPGES-1 in the microcirculation is consistent with an endothelial EP4-dependent mechanism. Here, loss of *Ep4* in the endothelium impaired microcirculatory perfusion in MI/R (Fig. 6g). This observation also explains the contrasting impacts on infarct size of global *Ep4*^21^ versus cardiomyocyte-specific *Ep4* gene deletion^22^, demonstrating the important contribution from the endothelium. Plasma levels of IL22 were unaltered (Supplementary Fig. 4C), suggesting that the protective effect of mPGES-1 in MI/R does not rely on IL22, a cytokine that restrains sepsis-related systemic inflammation downstream of PGE_2_^37^. Our study reveals a protective role of PGE_2_ in limiting acute sterile inflammation. The mechanism of mPGES-1 in protecting against MI/R injury is summarized in Fig. 8.

We found that COX-1, but not COX-2, is most likely to mediate the acute reperfusion injury in vivo (Fig. 1). Previous studies have shown that COX-2 mediates a late-phase, protective effect of pre-conditioning on the myocardium^15^ and it is required for chronic cardiac healing after sustained ischemia^38^. Thus, COX-1 may play a critical role in protecting the heart in acute myocardial infarction with reperfusion, whereas COX-2 may contribute to cardioprotection at a later phase of sustained ischemia, consistent with the association of heart failure with NSAIDs, both selective and non-selective for inhibition of COX-2^1^ (Fig. 8). This mechanism expands our current understanding of the cardiovascular risk of NSAIDs^39,40^.

Our previous studies in mice suggest that chronic inhibition of mPGES-1 is less likely than NSAIDs to cause thrombosis (e.g., myocardial infarction)^5^, and reduces restenosis after vascular injury, due to the increased biosynthesis of PGI_2_.^10,12^ However, in the setting of myocardial infarction, mPGES-1 inhibition may present a risk to patients undergoing reperfusion. It remains to be determined whether improving microcirculation via EP4 activation will benefit recovery in patients undergoing acute reperfusion.

## Methods

### Mice and drug administration

Mice deficient in *Cox-1*, *Cox-2*, or *mPges-1*, as described^5^, were obtained from the FitzGerald lab (University of Pennsylvania), with permission from original inventors. *Cox-1/2* deficient mice were maintained on a mixed C57B6 × 129/Sv genetic background. Knockout (KO) mice and littermate controls were generated from intercrossing heterozygous mice (or with homozygous KO mice). Given that no difference in the characterized phenotypes was detected between the heterozygous and wild-type littermates, these control mice were pooled together for analysis. The *mPges-1* KO mice were backcrossed to a C57B6 background for over ten generations. C57B6 wild-type (WT, *Ptges*^+/+^), or *Ptges*^+/−^ mice derived from breeding of *Ptges*^+/−^ with *Ptges*^+/−^ (or^−/−^) were used as controls for the *mPges-1* KO mice. Endothelial-specific *Ep4* (*Ptger4*) KO mice were created by crossing *Ptger4*-floxed mice (*Ptger4*^*f/f*^) with *Cdh5*-promoter driven *CreERT2* (*Cdh5 (PAC)-CreERT2+*) mice, as described^12^. The resulting endothelial *Ep4* conditional-knockout mice (*Ptger4*^*f/f*^ *Cdh5-CreERT2+*: abbreviated, EC *Ep4* cKO, or cKO) and littermate controls (*Ptger4*^*f/f*^ *Cdh5-CreERT2*−, Ctl) were postnatally treated with tamoxifen to induce endothelial *Ep4* deletion. Inducible global *Ep4* KO mice were similarly prepared with used of R26CreER mutant mice from the Jackson lab, which have a tamoxifen-inducible CreER driven by the endogenous mouse Gt(ROSA)26Sor promoter. In all cases, transgenic mice deficient in the indicated gene were compared with age-, and sex-matched control mice.

Celecoxib (100 mg/kg) or vehicle (1% methyl cellulose) was orally gavaged before MI/R surgery and again 20 h after MI/R. For the misoprostol study, male C57B6 mice (8–10 weeks old) were subjected to left coronary artery ligation and randomized to receive 50 or 100 μg/kg misoprostol or vehicle (saline) via i.p. injection after ligation and at 4, 8, 12 h after reperfusion. Mice were euthanized 24 h after reperfusion for sample collection.

For assessment of EP4 antagonism, 8-week-old mice were subjected to MI/R surgery and treated by intraperitoneal injection of CAY10580 (a selective EP4 agonist, Cayman Chemical, Ann Arbor, USA; 200 μg/kg body weight, twice a day), L-161,982 (an EP4 selective antagonist, Sigma-Aldrich, USA; 5 mg/kg body weight, once a day), or vehicle (2% ethanol/DMSO in PBS) immediately after ligation.

All animal protocols complied with all relevant ethical regulations and were approved by the Institutional Animal Care and Use Committee, the Experimental Animal Center, Fuwai Hospital, National Center for Cardiovascular Diseases, China. The experimental performers were blind to genotype/treatment grouping information during the experimental conduction and quantification.

### Myocardial ischemia/reperfusion model

Induction of myocardial I/R injury was conducted using a method without artificial ventilation^41^. Briefly, mice were anesthetized with 3% isoflurane inhalation followed by 1.5–2% isoflurane inhalation for anesthesia maintenance. The mice were placed in a supine position. The skin on the left thorax was slit, and the thoracic muscle was simply separated. Then, the thoracic cage was promptly exposed via thoracotomy through the 4th intercostal space on the left. After the pericardium was opened, the mouse heart was exposed, and the left anterior descending (LAD) coronary artery was ligated with a slipknot at ~3 mm from the origin using a 7–0 silk suture. The success of the ligation was confirmed by the anterior wall of the LV turning pale coincident with ST-segment elevation on the electrocardiogram (ECG). The heart was then quickly placed back into the thoracic space, followed by manual evacuation of air and the chest closed with a 4–0 suture. The internal end of slipknot suture was cut as short as possible, and the other end of the suture was ~0.8 cm long and remained outside of the chest. Anesthesia was then stopped, and the animals were allowed to recover. After 30 min of ischemia, mice were re-anesthetized, the slipknot was released by pulling the long end of the suture smoothly and gently until a feeling of release was sensed, at which time, the myocardial reperfusion began. The suture was removed to avoid prolonged tissue injury. At 24 h post I/R, cardiac function and the ventricular structure were determined via echocardiography (VisualSonics VeVo 2100 Imaging System) by evaluating the ejection fraction (EF), left ventricular fractional shortening (FS), and left ventricular anterior wall thickness [LVAW; s(systolic)/d(distolic)]. Mortality was similar across the groups, at ~20%. At 24 h post I/R, the LAD was reoccluded in the previous position, and 2% Evans blue dye (Sigma, Darmstadt, Germany) was injected into the heart cavity through the ascending aorta. The mouse was then euthanized, and its heart was harvested and rinsed in PBS. The heart was then frozen at −80 °C for 30 min and cut transversely into six slices below the ligature. The slices were incubated with 1% 2,3,5-triphenyltetrazolium chloride (TTC, Amresco, USA) at 37 °C for 10 min in the dark room and then fixed with 10% formalin for 2 h. A stereomicroscope (Zeiss, Germany) was used to take pictures. The areas of the ischemic region, infarcted tissue, and LV were measured and calculated using the Image-Pro Plus 6.0 software (Media Cybernetics).

### Detection of microcirculatory perfusion

To determine microcirculatory blood flow during myocardial (M) I/R, mice were anesthetized using pentobarbital sodium, intubated and ventilated with a positive-pressure respirator. After removing the thoracic muscle, the thoracic cage was opened via thoracotomy through the 4th intercostal space on both sides and the midsternum. The heart was then completely exposed by cutting off 2–4 ribs on both sides and removing the anterior thorax wall. To induce heart ischemia, the LAD coronary artery was ligated with a 7–0 silk suture 2–3 mm from the origin. Thirty minutes later, the ligature was removed to allow reperfusion of the ischemic myocardium. To monitor microcirculatory perfusion, hearts were scanned using laser Doppler flowmetry (PeriCam PSI System, Perimed, Sweden) at baseline (prior to ligation), 0 and 30 min post ligation, and 0, 10, 20, and 30 min post reperfusion. Left ventricular epicardial blood flow at the ischemic part was determined and analyzed blindly.

To measure microcirculatory response to hindlimb I/R, mice were anesthetized by pentobarbital sodium, and the left femoral artery was exposed with blunt dissection. Hindlimb ischemia (HLI) was induced via ligating the artery at the proximal site of the femoral artery bifurcation, followed by reperfusion via releasing the suture 10 min later. Blood flow in the distal extremity of hindlimb during the I/R was determined by PeriCam PSI System before and immediately after the ligation and at different time points after reperfusion. For misoprostol study, mice were injected i.p. with misoprostol (100 μg/kg, in saline) 10 min before ischemia.

### Measurement of vascular response in hindlimb I/R

Mice (18–20 weeks) were anesthetized by i.p. injection of pentobarbital sodium (80 mg/kg). The mice were fixed in a supine position. Then the arterioles downstream of the femoral artery were gently exposed. Mice were injected i.v. via angular vein with Rhodamine 6G (Sigma, Darmstadt, Germany; 5 mg/kg in saline) or with Rhodamine 6G plus an antagonist of EP1 (ONO-8130^42^, Ki = 1.9 nM, MW = 500.6, Cayman, USA), EP2 (PF-04418948^43^, IC50 = 16 nM, MW = 409.4, Selleck, USA), EP3 (L-798106^44^, Ki = 0.3 nM, MW = 536.4, Cayman, USA), or EP4 (GW627368X^45^, Ki = 100 nM, MW = 544.6, MedChem Express, USA), all at 2.5 mg/kg (in saline with 10% DMSO, V/V), ~2 min before imaging. One arteriole with diameter between 15 and 25 μm was randomly chosen for measuring vascular reactivity under a Nikon microscope (E400, Japan). The mouse position was then fixed, and hindlimb ischemia was induced by applying a rubber band tie around the ipsilateral thigh, and ischemia was verified by complete blockade of blood flow verified with the microscope (excited with green fluorescence). Ten minutes later, reperfusion was induced by release of the rubber band. The microvessel response was videotaped with a JPLY camera at a magnification of 200 ×. Customized image analysis software was used to assess the vessel diameter. The vessel diameter was measured prior to ligation, 10 min post ligation, and, 1 (for some experiments), 2, 5, 10, 15 (for some experiments), 20 and 30 min post reperfusion.

### Isometric vessel tension study

After mice were sacrificed by overdosing of pentobarbital sodium (100 mg/Kg), aortas and the second order branches of mesenteric arteries were isolated for vessel tension studies^46^. Briefly, the arteries were cut into vessel rings, with each ring ∼2 mm in length, in ice-cold Krebs-Ringer bicarbonate solution (118.3 mM NaCl, 4.7 mM KCl, 2.5 mM CaCl_2_, 1.2 mM MgSO_4_, 1.2 mM KH_2_PO_4_, 25 mM NaHCO_3_, and 11.1 mM glucose). The rings were suspended in the chambers of a multi myograph (620 M, Danish, Myo Technology A/S, Aarhus, Denmark) using two tungsten wires. Each chamber contained 5 mL Krebs solution which was maintained at 37 °C and constantly bubbled with 95% O_2_ and 5% CO_2_. At the beginning of the experiments, each vessel ring was brought to its optimal tension (∼2.5 mN for aortas, ∼1 mN for mesenteric arteries). After 60 min equilibration, the arteries were firstly contracted with 60 mM KCl, followed by three washes with warmed Krebs solution. To determine the endothelium-dependent vasodilation, the vessels were contracted with 3 µM phenylephrine (PE), followed by adding acetylcholine (ACh) at different concentrations (0.003–10 μM). The vessels were then washed three times again, and L-NAME (100 μM) and indomethacin (10 μM) were added to inhibit endothelial cell activities. After 30-min incubation with L-NAME and indomethacin, the endothelium-independent vasodilation was examined by determining vasodilation of sodium nitroprussiate (SNP, a NO donor) to U46619 (100 nM) or PE (3 µM).

### Intravital microscopy of leukocyte adhesion

Leukocyte adhesion was monitored in vivo by intravital microscopy of the femoral artery. Mice were injected i.v. via angular vein with Rhodamine 6G (Sigma, Darmstadt, Germany; 5 mg/kg in saline) before femoral artery ligation. Leukocyte adhesion was live-imaged and recorded with a Nikon microscope (E400; excited with green fluorescence) that was connected to a Nikon camera (S1TC01M). Time-lapse video was analyzed offline by a technician blind to mouse grouping information, the number of rolling leukocytes was counted for 1 min at different time points (prior to ligation; 2, 5, 10, 20, and 30 min post reperfusion).

### Bone marrow transplantation

Briefly, recipient mice (WT or *mPges-1* KO) were irradiated with one sub-lethal dose of 10 Gy (Gammacell 40 ^137^Cs γ-irradiation source) for a period of 10–12 min. Each recipient was reconstituted via retro-orbital vein injection of 5 × 10^6^ WT or KO bone marrow cells. Mice were then allowed to recover for ~5 weeks, including 1–2 weeks on antibiotic treatment (1–2 mg/ml gentamycin or neomycin 1.1 mg/ml and polymyxin B sulfate 1000 U/ml in drinking water). The bone marrow chimeric mice were then subjected to MI/R injury study.

### Analysis of cardiac neutrophils by flow cytometry

Hearts were isolated 24 h post MI/R, and about 20 mg of heart tissue was cut from the apex. Minced tissue was then digested with collagenase (0.895 mg/ml, Type II, Sigma, USA) and protease (0.5 mg/ml, Type XIV, Sigma, USA) in a 37 °C shaker for 7 min. The digestion mix was filtered using a 74-µm strainer and centrifuged at 200 × *g* for 5 min at 4 °C. The cell pellet was resuspended by 100 µl FACS buffer, 50 µl was stained with an antibody mixture (PE Rat Anti-Mouse CD45,1:50, 553081; FITC Rat Anti-Mouse CD11b, 1:50, 553310; APC-Cy^TM^ 7 Rat Anti-Mouse Ly-6G,1:50, 560600; BD Biosciences; New York, USA) for 30 min on ice in dark. Then each sample was added with 250 µl FACS buffer, filtered with a 74 µm nylon membrane and analyzed by flow cytometer at a constant flow rate and a fixed collection time.

### Cell study

Myeloid cell isolation: C57B6 mice were injected intra-peritoneally with 4% Brewer modified Thioglycollate medium (BD, Franklin Lakes, NJ, USA) (1 mL/mouse). After 5 h, peritoneal ascites was washed out with PBS containing 0.1% BSA for collection, followed by centrifugation (200 × *g*, 3 min). The sedimentary leukocytes, which were mainly myeloid cells, were harvested and used for cell adhesion study.

Blood leukocyte isolation: Human or mouse whole blood were lysed in red blood cell lysis buffer (BD, Franklin Lakes, NJ, USA) for 5 min at room temperature, followed by centrifugation (1000 × *g*, 2 min). Cell pellets (leukocytes) were resuspended in saline for experiments.

Mouse neutrophil isolation: Mice were anesthetized by intraperitoneal injection of pentobarbital sodium (80 mg/kg body weight). Blood was collected by retro-orbital bleeding; 1 ml of blood was mixed with 7 ml HBSS (Corning, New York, USA) containing 15 mM EDTA, followed by 500 × *g* centrifugation for 10 min, at room temperature. The cell pellet was lysed by mixing with 5 ml BD red blood lysis buffer at RT for 5 min, followed by adding 5 ml RPMI (Gibco, Waltham, MA, USA) media containing 2% FBS. After 10-min centrifugation at 200 × *g*, the cell pellet was washed once with isolation buffer (HBSS with 2 mM EDTA and 1% BSA) and then centrifuged at 400 × *g* for 5 min, at room temperature. The cell pellet was gently resuspended in 6 ml 45% Percoll (GE, New York, USA), loaded onto Percoll gradient (81 −62 −45%), and centrifuged at 1500 × *g* for 30 min with break off. The neutrophil layer was collected to 10 ml isolation buffer. After 10 min centrifugation at 1200 × *g*, cells were resuspended in 5 ml RPMI (containing 10% FBS).

Human neutrophil isolation: Human neutrophils were freshly prepared at room temperature from healthy donors after obtaining informed consent. Neutrophils were isolated using Lymphocyte-poly isolation media (Cedarlane Labs, Ontario, Canada) from EDTA-anticoagulated peripheral blood. Briefly, blood was loaded to the separation media at a ratio of 1:1 in volume in a conical tube, and was centrifuged at 500 × *g* for 35 min with the break off. The neutrophil layer was collected into 40 ml of PBS without Ca^2+^ and Mg^2+^, and centrifuged at 400 × *g* for 10 min. The pellet was resuspended in 1 ml PBS, subjected to red blood cell lysis buffer, and was then centrifuged at 200 × *g* for 10 min. Cell pellet was washed in PBS and resuspended in PBS.

Cell adhesion study: Endothelial cells were seeded and cultured in a 96-well flat bottom plate to reach full confluence. The cells were treated with indicated drugs (dissolved in DMSO) in a medium containing 5% FBS for 2 h, except for mPGES-1 inhibitor (Cay10526), which was incubated for 12 h. Then, complement factor 5a (C5a) in PBS was added to a final concentration of 100 nM, and incubated for 30 min. Peritoneal or blood leukocytes, prepared in RPMI medium containing 10% FBS, were then loaded to the EC monolayer culture, and incubated for another 30 min. The coculture was washed three times in RPMI. The adherent leukocytes were then counted in a field of view at the center of each well under Leica microscope (dmi4000B; excited with green fluorescence) at ×100 magnification.

The studies using human whole blood and cells complied with all relevant ethical regulations. Ethical approval was obtained from the Institutional Review Board, Fuwai Hospital, National Center for Cardiovascular Diseases, China. Informed consent was obtained from all human participants.

### Immunofluorescence staining

Hearts were collected, frozen, embedded and sectioned at a thickness of 10 μm. For each sample, the sections were serially gathered at 200 μm intervals below the ligature for a total of four levels. Tissue sections were fixed with 95% ethanol for 15 min, and then incubated at room temperature for 90 min, with a goat serum containing 0.3% Triton X-100 for blocking and membrane rupture. The heart sections were incubated with rabbit anti-myeloperoxidase (MPO) antibody (1:50; Abcam, ab9535, Cambridge, UK) at 4 °C overnight, rinsed with PBST, and then incubated with Alexa Fluor 488 goat anti-rabbit antibody (1:200; ZSGB-BIO, ZF-0511, Beijing, China) for 1 h at 37 °C. For dual staining, the heart sections were incubated at 4 °C overnight in a mixture of sheep anti-Von Willebrand factor (VWF) antibody (1:100; Abcam, ab11713, Cambridge, UK, for endothelial cell delineation) and rabbit antibody against EP1 (101740), EP2 (101750), EP3 (101760), or EP4 (101775). All EP1-4 antibodies were from Cayman (Michigan, USA) and used at a dilution of 1:200. The secondary antibodies used were FITC 488 goat anti-rabbit antibody and Alexa Fluor 594 donkey anti-sheep IgG (1:200; Invitrogen, A-11016, California, USA). Coverslips were mounted with a VectaShield medium containing DAPI to stain nuclei. The sections were imaged using a Zeiss inverted fluorescence microscope (AXI0; Zeiss, Germany) equipped with a Zen software. MPO-positive cells were counted for 4 levels of each sample using the Image-Pro Plus 6.0 software (Media Cybernetics, Inc., Rockville, MD, USA).

### ATP and ADP measurement

Heart tissue samples at area of risk or that from matching part of naive animals were harvested. ADP and ATP levels were measured using colorimetric/fluorometric assay kit for ATP (MAK190, SIGMA, CA, USA) and ADP (MAK081, SIGMA, CA, USA), according to the manufacturer’s instructions. Protein concentration of cell lysate was determined using BCA protein assay. ATP abundance was normalized to tissue weight.

### Analysis of MPO concentrations

Heart tissue samples at area of risk or that from matching part of naive animals were harvested. About 10 mg tissue of each sample was lysed by adding 100 µl lysis buffer (200 mM NaCl, 5 mM EDTA, 10 mM Tris, 10% glycerin, 1 mM PMSF, 1 µg/ml leupeptin and 28 µg/ml aprotinin, PH 7.4). After homogenization, sample was centrifuged twice (1500 × *g*, 4 °C, 15 min) to avoid contamination of cell debris. The level of MPO was then determined spectrophotometrically by commercial MPO ELISA Kit (HK210, Hycult Biotechnology, Uden, Netherlands) according to the manufacturer’s instruction. MPO concentration was normalized to tissue weight.

### RT-PCR

Cellular RNA was extracted by TRIzol (Invitrogen, California, USA) according to the manufacturer’s instruction. PrimeScript™ RT Master Mix (TaKaRa, Tokyo, Japan) was used to prepare cDNA. Quantitative RT-PCR was performed using TransStart Tip Green qPCR SuperMix (TransGen, Beijing, China). The sequences of the primers used were as follows: *β-actin*, ACCTTCTACAATGAGCTGCG (forward) and CTGGATGGCTACGTACATGG (reverse); *Ep4*, GCCATCAGGATTGCTTCT (forward) and ACCAACAGGACACTCTCA (reverse); *Cox-1*, CACTCGCCTCATCCTTATAG (forward), GTTCCTACCTCCACCAATC (reverse); *Cox-2*, CCTTCTCCAACCTCTCCTA (forward), ACACCTCTCCACCAATGA (reverse).

### Prostanoid determination

PGE_2_, was determined by liquid (L) chromatography (C)–tandem mass (M) spectrometry (S)^47^. UPLC BEHC18 column (1.7 μm, 100 × 2.1 mm i.d.) consisting of ethylene-bridged hybrid particles (Waters, Milford, MA) was used for Chromatographic separations. The isolates were analyzed using a 5500 QTRAP hybrid triple quadrupole linear ion trap mass spectrometer (AB Sciex, Foster City, CA) equipped with a turbo ion spray electrospray ionization source. EDTA-anticoagulated plasma samples were harvested 24 h post MI/R surgery and used for PGE_2_ determination. Serum-free cell culture media was used to culture primarily isolated endothelial cells or neutrophils, for indicated duration of time. After 5 min centrifugation at 6000 × *g*, supernatant was stored at −80 °C for PGE_2_ determination.

Twenty-four hours urine was collected from mice subjected to MI/R injury. Biosynthesis of PGE_2_ and PGI_2_ was estimated via measuring their urinary metabolites, tetranor-PGEM and 2,3-dinor-6-keto-PGF_1α_, respectively, by liquid chromatography–tandem mass spectrometry^48^.

### IL-22 measurement

Plasma IL-22 was measured with mouse IL-22 Quantikine ELISA Kit (R&D Systems, Minnesota, USA), following the manufacturer’s instruction.

### Modified miles assay

Briefly, *mPges-1* WT and KO mice were subjected to coronary artery ligation for 30 min, and 1% Evans blue (200 μL) was injected intravenously by retro-orbital injection before reperfusion^49^. Mice were euthanized 4 h after reperfusion and perfused through the aorta with citrate buffer (pH 4). The area at risk of left ventricular (below the ligation) including ventricular septum was dissected, and Evans blue was extracted in 1 mL of formamide for 18 h at 70 °C. After centrifugation, absorbance was measured at 620 nm by using a spectrophotometer. The amount of extravasated Evans blue (μg) was determined from a standard curve and normalized to heart tissue weight (g).

### Statistical analysis

Statistical analysis was performed using GraphPad Prism 5 software (GraphPad Software Inc., San Diego, California, USA). Student’s *t*-test (two-tailed, unpaired) was used for two-group comparisons. When a time factor was involved, two-way ANOVA was used for data comparisons. Multiple-group comparisons were made using one-way ANOVA. Following either one-way or two-way ANOVA, post hoc tests were performed with Bonferroni correction unless otherwise indicated in figure legends. Data were expressed as mean ± standard error of mean (SEM). Differences were considered statistically significant at *P* < 0.05. Statistical significance is demarked as *P < 0.05, **P < 0.01, ***P < 0.001.

### Reporting Summary

Further information on experimental design is available in the Nature Research Reporting Summary linked to this article.

## Supplementary information

Supplementary Information

Reporting Summary

## Data Availability

The data that support the findings of this study are available from the authors upon reasonable request. See Author contributions.
