# Supplementary material for: The cyclooxygenase-1/mPGES-1/endothelial prostaglandin EP4 receptor pathway constrains myocardial ischemia-reperfusion injury
Source: Nat Commun. 2019 Apr 23;10:1888. doi: 10.1038/s41467-019-09492-4 (PMC6478873; doi:10.1038/s41467-019-09492-4)
Supplement: Supplementary file 1 — Supplementary Information [file 41467_2019_9492_MOESM1_ESM.pdf]

Supplementary Material

The Cyclooxygenase-1/mPGES-1/Endothelial Prostaglandin EP4 Receptor Pathway Constrains Myocardial Ischemia-Reperfusion Injury

Zhu et al.

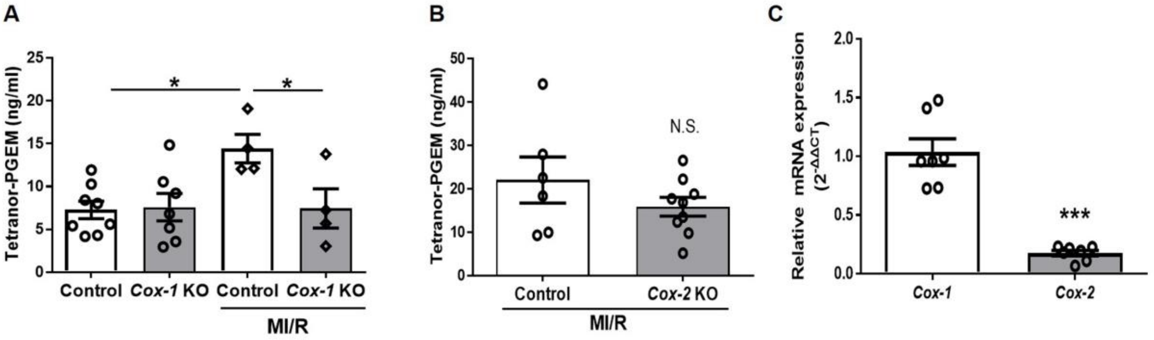

Supplementary Figure 1. Urinary levels of PGE metabolite and cardiac expression of COXs.

Urinary levels of the major metabolite of PGE_2, tetranor-PGE metabolite (PGEM), were determined by liquid chromatography–tandem mass spectrometry for *mice* deficient in *Cox-1* (**A**, n=8,7,4,4) or *Cox-2* (**B**, n=6 Control, 9 KO, p=0.39). Expression of *Cox-1* and *Cox-2* was detected in hearts from WT *mice* by quantitative PCR (**C**, n=7). MI/R denotes myocardial ischemia/reperfusion. A: One-way ANOVA with Tukey's multiple comparison test. B,C: Unpaired Student's t test. Error bar indicates Standard Error of Mean.

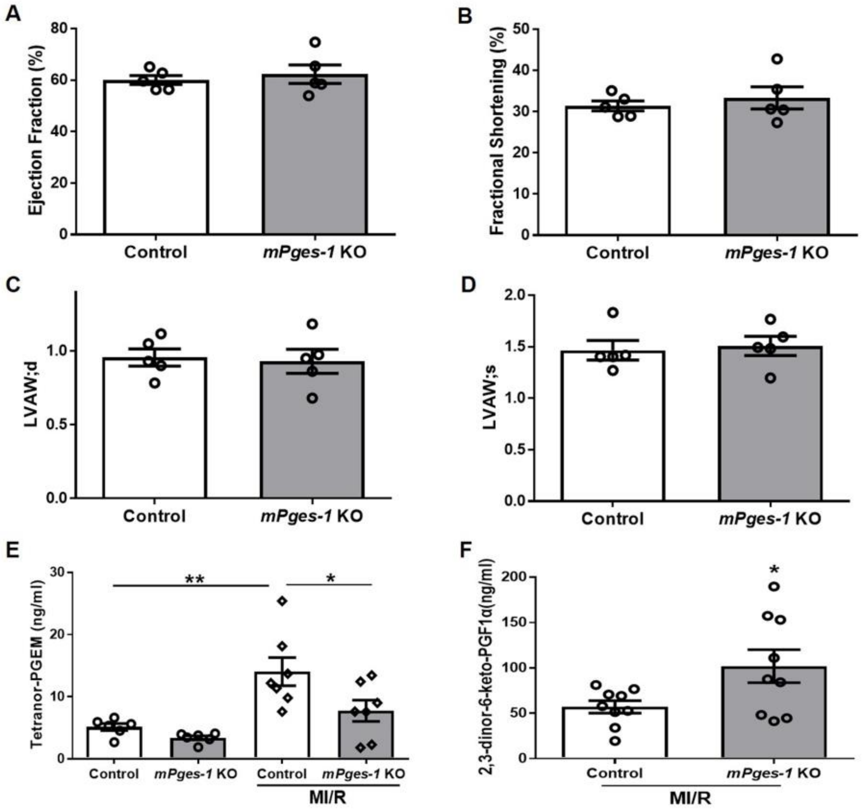

Supplementary Figure 2. Baseline ultrasonographic parameters and urinary levels of PG metabolites.

No difference in the examined ultrasonographic parameters was detected between naive *mPges-1* KOs and littermate controls (**A-D**, n=5, p>0.05). Urinary levels of tetranor-PGEM (**E**, n=6,6,7,7 females) and PGI_2 (2,3-dinor-6-keto-$PGF_{1\alpha}$, **F**, n=9 Control, 10 KO) were detected in *mice* subjected to MI/R. A-D, F: Unpaired Student's t test, E: One-way ANOVA with Bonferroni multiple comparison test. Error bar indicates Standard Error of Mean.

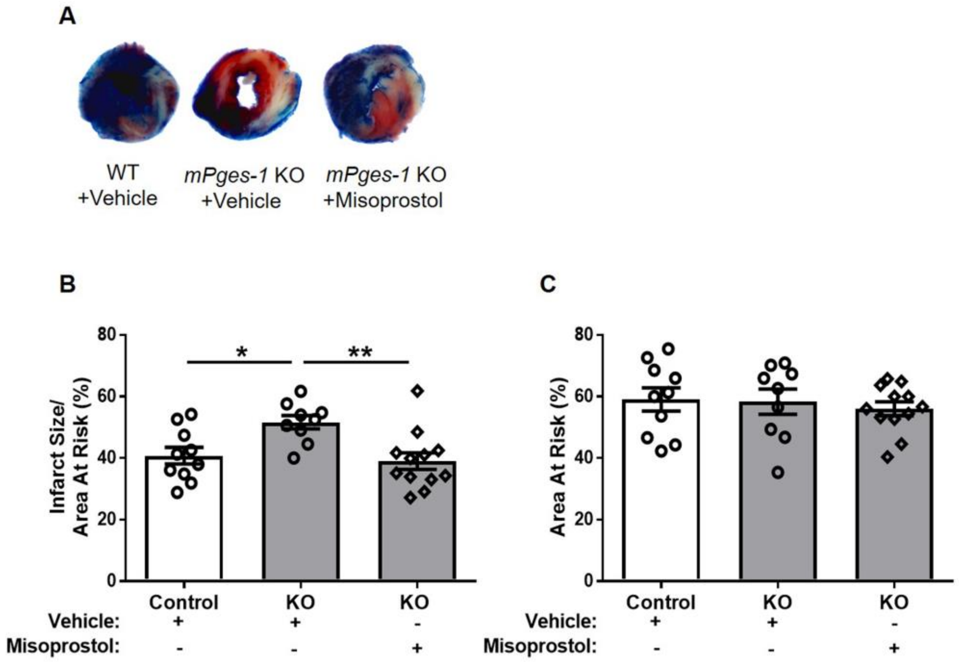

Supplementary Figure 3. Misoprostol treatment attenuated the exaggerated MI/R injury in *mPges-1* KOs.

Mice were treated with 100 μg/kg misoprostol via i.p. injection following LAD ligation before reperfusion, and additionally at 4, 8, 12 hours of reperfusion. (**A**) Representative photographs of TTC stained sections from Evans blue perfused hearts. Infarct size (**B**) and AAR (**C**) were quantified. One-way ANOVA with Tukey's multiple comparison test, n=10,9,12. Error bar indicates Standard Error of Mean.

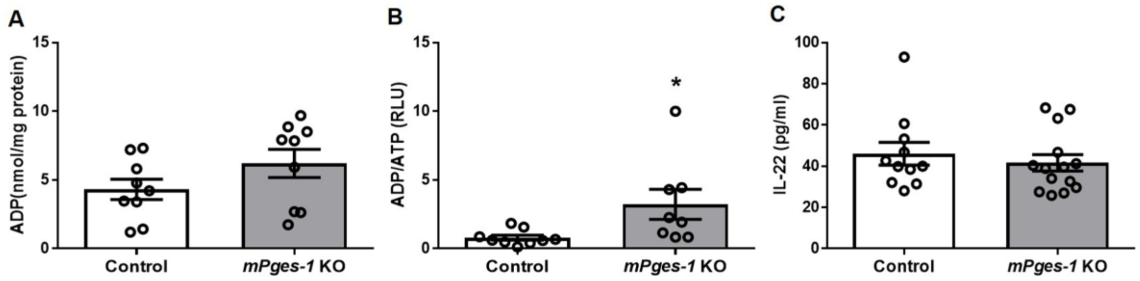

Supplementary Figure 4. Heart levels of ATP metabolites and plasma levels of IL22.

Heart and plasma samples were harvested at 24 hours post MI/R from *mPges-1* KO and littermate controls for ATP metabolites (**A**,**B**, n=9) and IL22 (**C**, n=14 Control, 11 KO, p=0.513) detection, respectively. Unpaired Student's t test. Error bar indicates Standard Error of Mean.

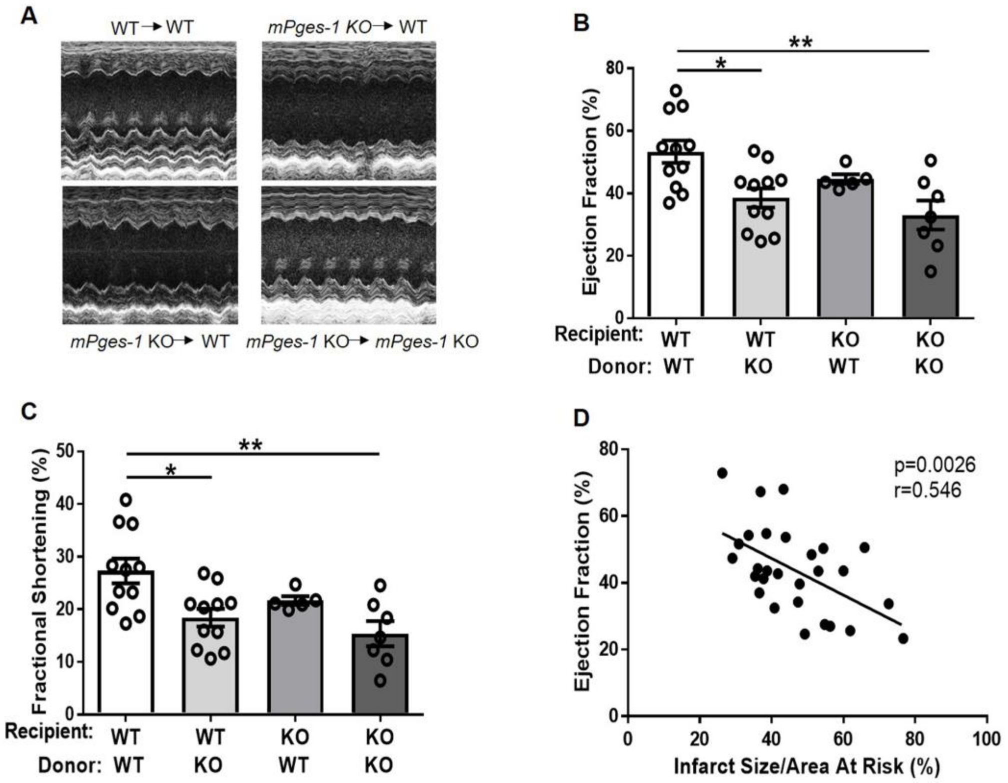

Supplementary Figure 5. Cardiac ultrasonography of *mPges-1* chimeric *mice* after MI/R injury.

Representative echocardiograph (**A)**, ejection fraction (EF)(**B)** and fractional shortening (FS) (**C**) of chimeric *mice* (B&C, n=11,11,5,7) were shown. The correlation between left ventricular ejection fraction and infarct size was analyzed using linear regression in the chimeric animals (**D**, n=28, r=0.546, p=0.0026). Arrow connects donor *mice* with recipient *mice*. B,C: One-way ANOVA with Tukey's multiple comparison test. Error bar indicates Standard Error of Mean.

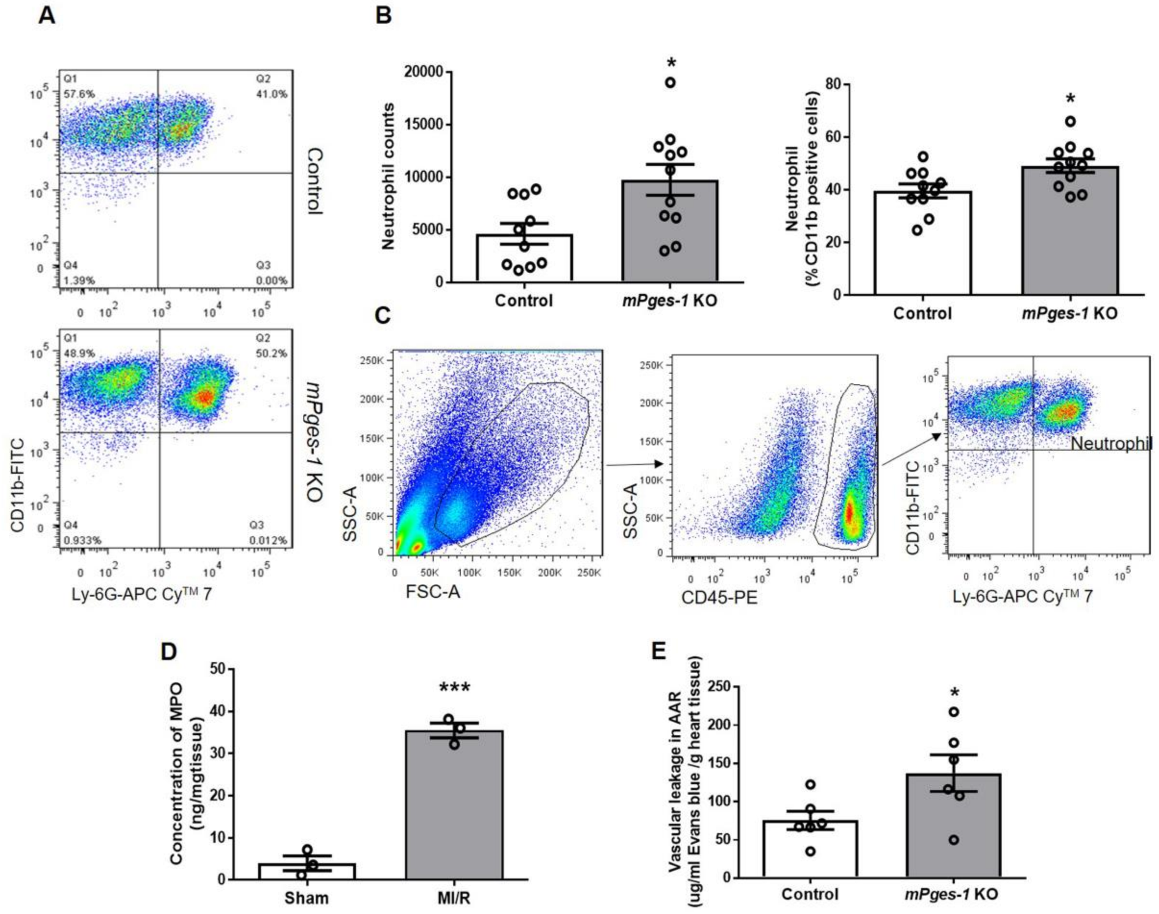

Supplementary Figure 6. *mPges-1* KO increased myeloid cell infiltration and vascular permeability in MI/R.

Ischemic cardiac tissue samples harvested at 24h post MI/R were dissociated to prepare for cell suspension and analyzed by flow cytometry(**A**). Absolute counts of neutrophils (CD11b+Ly6G+) and percent of neutrophils in $CD11b^+$ cells (**B**) were compared (n=10 Control, 11 KO). (**C**) Gating strategy to identify neutrophil (CD45+CD11b+Ly6G+) cells from *mPges-1* WT, KO and C57BL/6 WT *mice* by flow cytometry presented on Figure 7F, Supplementary Figure 6A. MPO concentration in heart samples was measured in WT *mice* between sham and MI/R groups (**D**, n=3), normalized by total protein content. Evans blue was extracted from ischemic heart and quantified for *mPges-1* KO and control *mice* vascular permeability after MI/R (**E**, n=6). See Method for details. Unpaired Student's t test. Error bar indicates Standard Error of Mean.

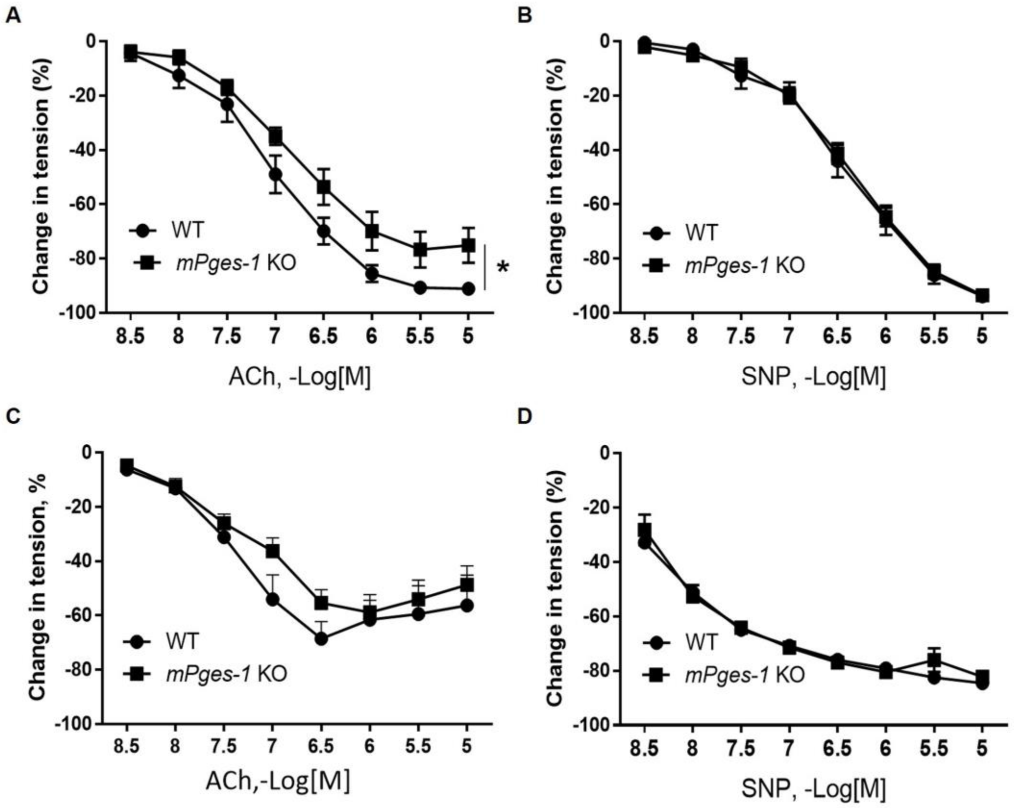

Supplementary Figure 7. *mPges-1* KO impairs acetylcholine-induced vasorelaxation in resistant mesenteric arteries but not in aortas.

Endothelium-dependent vasorelaxation to acetylcholine (ACh) was determined in the resistant mesenteric arteries (**A**, n=8 vessels) and aortas (**C**, n=10 vessels). After the endothelial cells were inhibited by L-NAME and indomethacin, endothelium-independent vasorelaxation to sodium nitroprussiate (SNP) was determined in the mesenteric arteries (**B**, n=8 vessels) and aortas (**D**, n=6 vessels). *P<0.05, two- way ANOVA. Error bar indicates Standard Error of Mean.

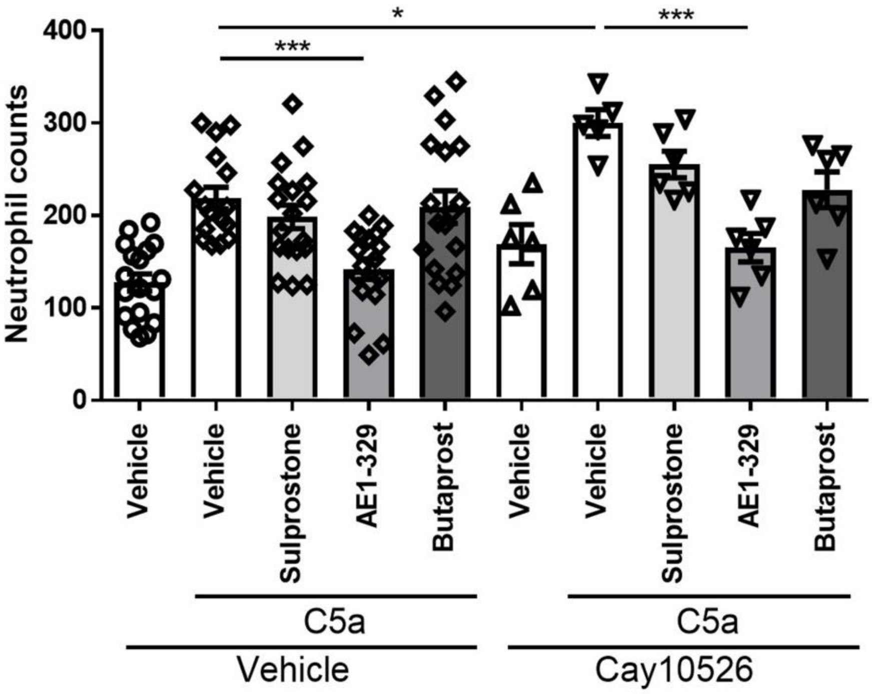

Supplementary Figure 8. Effects of mPGES-1 inhibition and PGE receptor activation in neutrophil adhesion to ECs.

Human microvascular ECs cultured as monolayer were treated with indicated drugs, followed by adding freshly prepared human blood neutrophils. Number of adherent cells was quantified 30 minutes later as detailed in the Methods. Agonists for EP1/3 (Sulprostone), EP2 (Butaprost) and EP4 (AE1-329) were all at 1 μM, and examined with or without 10 μM Cay10526 treatment. One-way ANOVA with Bonferroni's Multiple Comparison Test, n=18,16,18,17,18,6,5,6,6,6. Experiments were repeated twice. Error bar indicates Standard Error of Mean.

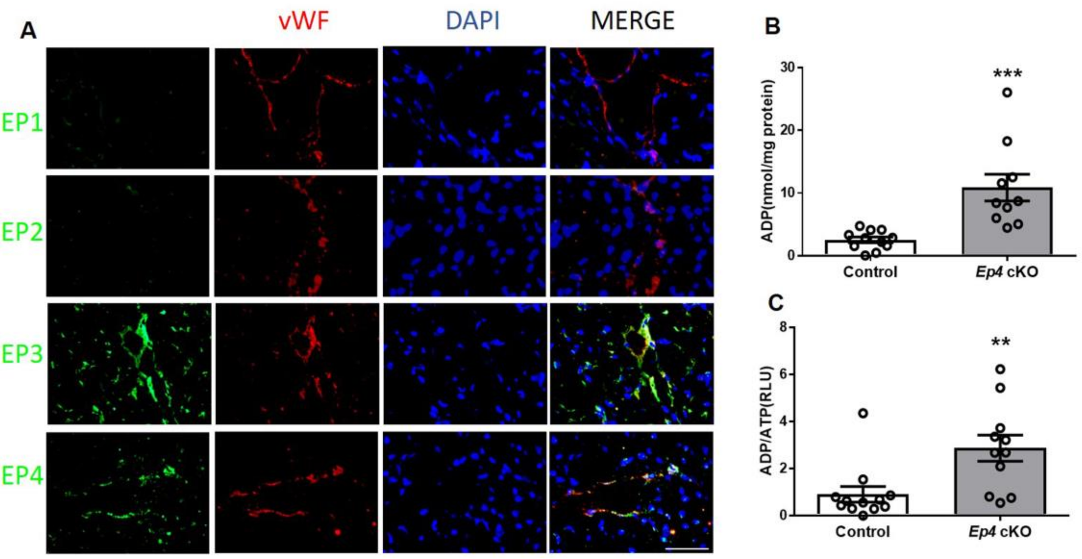

Supplementary Figure 9. Cardiac expression of EP1-4 and the level of ATP metabolites in the heart of *mice* underwent MI/R.

Hearts were isolated from WT *mice*, and tissue sections were stained for EP1-4 (green), VWF (red) and nuclei (DAPI, blue) by immunofluorescence staining. Representative graphs were shown (**A**). Hearts from EC *Ep4* KO and control *mice* were isolated 24 hours after MI/R, and the tissue levels of ADP, ATP/ADP ratios (**B**,**C**, n=11 Control, 12 cKO) were measured in the area of ischemic myocardium. Unpaired Student's t test. Scale bar is 50 μm. Error bar indicates Standard Error of Mean.

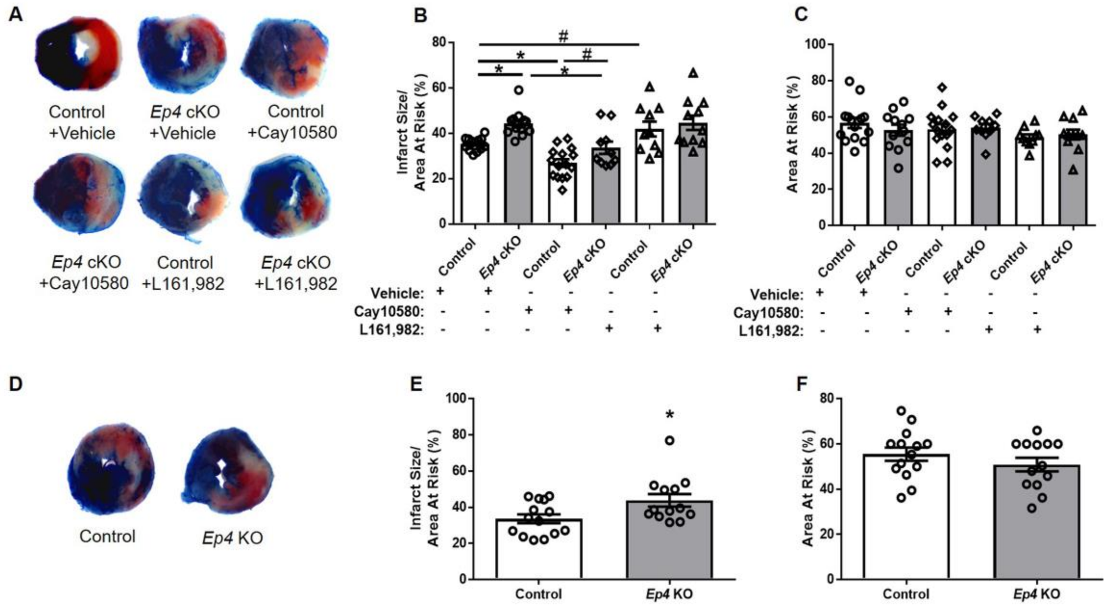

Supplementary Figure 10. Effects of EP4 agonism/antagonism/global deletion in MI/R injury.

EC *Ep4* cKO and littermate control *mice* were treated with Cay10580(an EP4 agonist) or L161,982(an EP4 antagonist) via i.p. injection following LAD ligation and before reperfusion. (**A**) Representative photographs of TTC stained sections from Evans blue perfused hearts were shown. Infarct size (**B**) and AAR (**C**) were quantified. Global *Ep4* KO *mice* were also subjected to MI/R injury. (**D**) Representative photographs of TTC/ Evans blue stained hearts sections were shown. Infarct size (**E**) and AAR (**F**) were quantified. One-way ANOVA with Tukey's multiple comparison test (B&C, n=15,12,15,10,10,11). Unpaired Student's t test (E&F, n=14 Control,13 KO). # denotes unpaired Student's t test for the indicated comparisons with p values <0.05. Error bar indicates Standard Error of Mean.

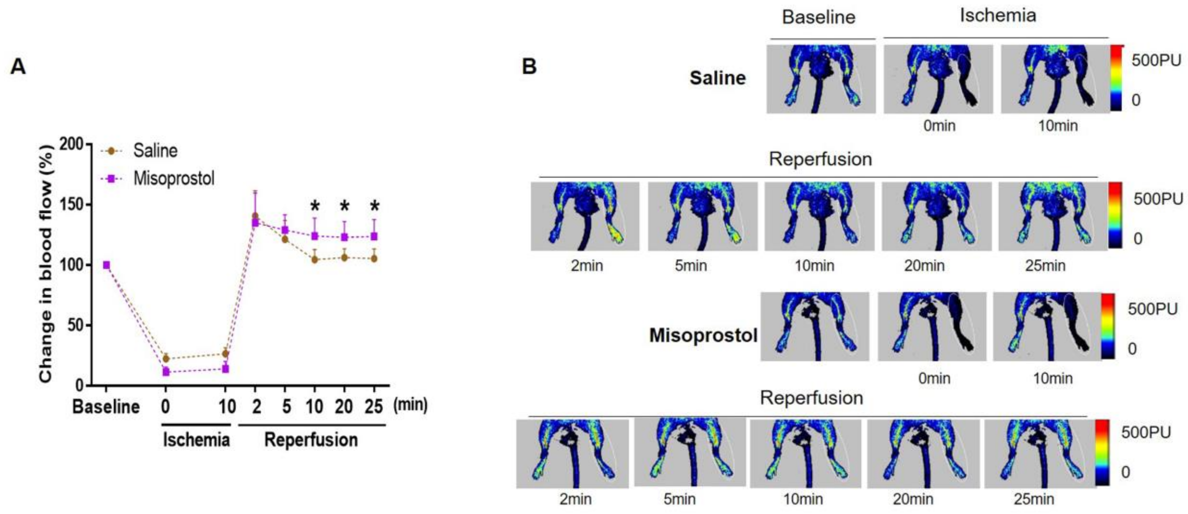

Supplementary Figure 11. Misoprostol improves hind-limb perfusion post I/R.
Mice were subjected to I/R at femoral artery. Microvascular flow relative to baseline was recorded at indicated time points before and after release of the artery ligation (**A**). Representative perfusion images at each timepoint were shown (**B**). Two-way ANOVA with Bonferroni's test, n=8,9. Error bar indicates Standard Error of Mean.
