# Supplementary material for: The cyclooxygenase-1/mPGES-1/endothelial prostaglandin EP4 receptor pathway constrains myocardial ischemia-reperfusion injury
Source: Nat Commun. 2019 Apr 23;10:1888. doi: 10.1038/s41467-019-09492-4 (PMC6478873; doi:10.1038/s41467-019-09492-4)
Supplement: Supplementary file 2 — Reporting Summary [file 41467_2019_9492_MOESM2_ESM.pdf]

# nature research

Corresponding author(s): Miao Wang

Last updated by author(s): Feb 28, 2019

# Reporting Summary

Nature Research wishes to improve the reproducibility of the work that we publish. This form provides structure for consistency and transparency in reporting. For further information on Nature Research policies, see Authors & Referees and the Editorial Policy Checklist.

Please do not complete any field with "not applicable" or n/a. Refer to the help text for what text to use if an item is not relevant to your study.
For final submission: please carefully check your responses for accuracy; you will not be able to make changes later.

## Statistics

For all statistical analyses, confirm that the following items are present in the figure legend, table legend, main text, or Methods section.

| n/a | Confirmed | |
|---|---|---|
| ☐ | ☒ | The exact sample size (*n*) for each experimental group/condition, given as a discrete number and unit of measurement |
| ☐ | ☒ | A statement on whether measurements were taken from distinct samples or whether the same sample was measured repeatedly |
| ☐ | ☒ | The statistical test(s) used AND whether they are one- or two-sided<br>*Only common tests should be described solely by name; describe more complex techniques in the Methods section.* |
| ☒ | ☐ | A description of all covariates tested |
| ☐ | ☒ | A description of any assumptions or corrections, such as tests of normality and adjustment for multiple comparisons |
| ☐ | ☒ | A full description of the statistical parameters including central tendency (e.g. means) or other basic estimates (e.g. regression coefficient) AND variation (e.g. standard deviation) or associated estimates of uncertainty (e.g. confidence intervals) |
| ☐ | ☒ | For null hypothesis testing, the test statistic (e.g. *F*, *t*, *r*) with confidence intervals, effect sizes, degrees of freedom and *P* value noted<br>*Give P values as exact values whenever suitable.* |
| ☒ | ☐ | For Bayesian analysis, information on the choice of priors and Markov chain Monte Carlo settings |
| ☒ | ☐ | For hierarchical and complex designs, identification of the appropriate level for tests and full reporting of outcomes |
| ☒ | ☐ | Estimates of effect sizes (e.g. Cohen's *d*, Pearson's *r*), indicating how they were calculated |

*Our web collection on statistics for biologists contains articles on many of the points above.*

## Software and code

Policy information about availability of computer code

| | |
|---|---|
| Data collection | No software was used. |
| Data analysis | Image Pro Plus 6.0, Graphpad Prism 5.0. |

For manuscripts utilizing custom algorithms or software that are central to the research but not yet described in published literature, software must be made available to editors/reviewers. We strongly encourage code deposition in a community repository (e.g. GitHub). See the Nature Research guidelines for submitting code & software for further information.

## Data

Policy information about availability of data

All manuscripts must include a data availability statement. This statement should provide the following information, where applicable:

- Accession codes, unique identifiers, or web links for publicly available datasets
- A list of figures that have associated raw data
- A description of any restrictions on data availability

The data that support the findings of this study are available from the authors upon reasonable request

# Field-specific reporting

Please select the one below that is the best fit for your research. If you are not sure, read the appropriate sections before making your selection.

☒ Life sciences ☐ Behavioural & social sciences ☐ Ecological, evolutionary & environmental sciences

# Life sciences study design

All studies must disclose on these points even when the disclosure is negative.

| | |
|---|---|
| Sample size | Sample size was chosen according to relevant references and experience. |
| Data exclusions | Individual data was excluded due to experimental abnormality was observed (e.g. mice showed clear sign of morbidity during the experiment process or were not successfully operated). |
| Replication | Cell experiments were conducted three times and were reproducible. Animal studies on infarct size using mPGES-1 KO, EC EP4 KO mice were repeated and reproduced in supplemental figures. Animal studies with drugs(such as agonist and antagonist) also confirmed the conclusion. |
| Randomization | Animals and cells were randomly allocated into experimental groups. |
| Blinding | The investigators were blinded to group allocation during data collection and analysis. |

# Reporting for specific materials, systems and methods

We require information from authors about some types of materials, experimental systems and methods used in many studies. Here, indicate whether each material, system or method listed is relevant to your study. If you are not sure if a list item applies to your research, read the appropriate section before selecting a response.

## Materials & experimental systems

| n/a | Involved in the study | |
|---|---|---|
| ☐ | ☒ | Antibodies |
| ☒ | ☐ | Eukaryotic cell lines |
| ☒ | ☐ | Palaeontology |
| ☐ | ☒ | Animals and other organisms |
| ☐ | ☒ | Human research participants |
| ☒ | ☐ | Clinical data |

## Methods

| n/a | Involved in the study | |
|---|---|---|
| ☒ | ☐ | ChIP-seq |
| ☐ | ☒ | Flow cytometry |
| ☒ | ☐ | MRI-based neuroimaging |

## Antibodies

Antibodies used

1. PE Rat Anti-Mouse CD45, BD Biosciences,catalog number:553081,clone number:30-F11, lot number:7138676;
2. FITC Rat Anti-Mouse CD11b,BD Biosciences, catalog number:553310,clone number:M1/70, lot number:4314772;
3. APC-Cy7 Rat Anti-Mouse Ly-6G,BD Biosciences,catalog number:560600,clone number:1A8, lot number:7352513;
4. Rabbit anti-myeloperoxidase (MPO) antibody ,Abcam, catalog number:ab9535, lot number:GR277624-1;
5. Sheep anti-Von Willebrand factor (VWF) antibody ,Abcam, catalog number:ab11713,lot number:GR307234-6;
6. Rabbit anti-EP1 antibody, Cayman ,catalog number:101740,lot number:0467177-1;
7. Rabbit anti-EP2 antibody ,Cayman ,catalog number:101750,lot number:0495947-1;
8. Rabbit anti-EP3 antibody ,Cayman ,catalog number:101760,lot number:0508042-1;
9. Rabbit anti-EP4 antibody ,Cayman ,catalog number:101775;
10. Alexa Fluor 488 goat anti-rabbit antibody ,ZSGB-BIO, catalog number:ZF-0511,lot number:132589;
11. Alexa Fluor 594 donkey anti-sheep IgG ,Invitrogen, catalog number:A-11016,lot number:1802764;

Validation

1. PE Rat Anti-Mouse CD45, Species:rat;Application: flow cytometry; Validation statement:The 30-F11 clone has been reported to react with all isoforms and both alloantigens of CD45, which is found on hematopoietic stem cells and all cells of hematopoietic origin, except erythrocytes; Relevant citations: Nat Med. 2000; 6(11):1212-1213; Figure used it in the study: Figure 7F, Supplemental figure 6A,E.
2. FITC Rat Anti-Mouse CD11b, Species:rat; Application: flow cytometry; Validation statement:The M1/70 monoclonal antibody specifically binds to CD11b, also known as Integrin alpha M (Itgam or αM); Figure used it in the study: Figure 7F, Supplemental figure 6A,E.
3. APC-Cy7 Rat Anti-Mouse Ly-6G, Species:rat; Application: flow cytometry; Validation statement:The 1A8 monoclonal antibody specifically binds to Ly-6G, a 21-25-kDa GPI-anchored protein; Relevant citations: J Immunol. 1993; 151(5):2399-2408; Figure used it in the study: Figure 7F, Supplemental figure 6A,E.
4. Rabbit anti-myeloperoxidase (MPO) antibody , Species:rabbit; Application: IHC-P, IHC-Fr, ICC/IF, IHC-FoFr; Myeloperoxidase isolated from human granulocytes.Relevant citations: J Clin Invest. 2018 Mar 1; 128(3): 916–930; Figure used it in the study: Figure 3C,6H,7H;
5. Sheep anti-Von Willebrand factor (VWF) antibody, Species:sheep; Application: IHC-Fr, Immunodiffusion, Flow Cyt, ELISA, ICC/IF; Immunogen: Purified human von Willebrand factor. Relevant citations: Arterioscler Thromb Vasc Biol. 2017 Feb;37(2):312-315; Figure used it in the study: Figure 6B,Supplemental figure 9A;
6. Rabbit anti-EP1 antibody, Species:rabbit; Application: IF,IHC,WB; Immunogen: Synthetic peptide from the C-terminal region of human EP1. Relevant citations: J. Biol. Chem.276(19), 16083-16091 (2001); Figure used it in the study: Supplemental figure 9A;
7. Rabbit anti-EP2 antibody, Species:rabbit; Application: IF,WB; Immunogen: Synthetic peptide from human EP2 receptor C-terminal. Relevant citations: J. Biol. Chem.276(19), 16083-16091 (2001); Figure used it in the study: Supplemental figure 9A;
8. Rabbit anti-EP3 antibody, Species:rabbit; Application: ICC,WB; Immunogen: Synthetic peptide from an internal region of

human EP3 receptor conjugated to KLH. Relevant citations: Physiol Rev. 1999 Oct;79(4):1193-226; Figure used it in the study: Supplemental figure 9A;
9. Rabbit anti-EP4 antibody, Species:rabbit; Application: ICC,IHC,WB; Immunogen: Synthetic peptide from the C-terminal region of human protein. Relevant citations: Physiol Rev. 1999 Oct;79(4):1193-226; Figure used it in the study: Figure 6B,Supplemental figure 9A;

## Animals and other organisms

Policy information about studies involving animals; ARRIVE guidelines recommended for reporting animal research

| | |
|---|---|
| Laboratory animals | 1. The COX-1 KO, COX-2 KO: maintained on a mixed C57B6 × 129/Sv genetic background.<br>2. The mPGES-1 KO mice: backcrossed to a C57B6 background for over 10 generations.<br>3. Endothelial-specific EP4 (Ptger4) KO mice: created by crossing Ptger4-floxed mice (Ptger4f/f) with Cdh5-promoter driven CreERT2 (Cdh5 (PAC)-CreERT2+) mice. Result: endothelial EP4 conditional-knockout mice (Ptger4f/f Cdh5-CreERT2+: abbreviated, EC EP4 cKO, or cKO) and littermate controls (Ptger4f/f Cdh5-CreERT2-, Ctl), postnatally treated with tamoxifen to induce endothelial EP4 deletion.<br>4. Inducible global EP4 KO mice: prepared with used of R26CreER mutant mice, which have a tamoxifen-inducible CreER driven by the endogenous mouse Gt(ROSA)26Sor promoter.<br>5. Sex: male and female were both used in the study.<br>6. Age: 8-16 weeks. |
| Wild animals | The study did not involve wild animals. |
| Field-collected samples | The study did not involve samples collected from the field. |
| Ethics oversight | All animal protocols complied with all relevant ethical regulations and were performed following the guidelines from the Animal Care and Use Committee, the Experimental Animal Center, Fuwai Hospital, National Center for Cardiovascular Diseases, China. |

Note that full information on the approval of the study protocol must also be provided in the manuscript.

## Human research participants

Policy information about studies involving human research participants

| | |
|---|---|
| Population characteristics | Participants involved in the study were all healthy male, with age ranged from 25-30. |
| Recruitment | Volunteer participants were recruited through the recruitment notice with informed consent. |
| Ethics oversight | The studies using human whole blood and cells complied with all relevant ethical regulations. Ethical approval was obtained from the Institutional Review Board, Fuwai Hospital, National Center for Cardiovascular Diseases, China. Informed consent was obtained from all human participants. |

Note that full information on the approval of the study protocol must also be provided in the manuscript.

## Flow Cytometry

### Plots

Confirm that:

☒ The axis labels state the marker and fluorochrome used (e.g. CD4-FITC).

☒ The axis scales are clearly visible. Include numbers along axes only for bottom left plot of group (a 'group' is an analysis of identical markers).

☒ All plots are contour plots with outliers or pseudocolor plots.

☒ A numerical value for number of cells or percentage (with statistics) is provided.

### Methodology

| | |
|---|---|
| Sample preparation | Mice were euthanized and hearts were isolated 24 hours post MI/R, and about 20 mg of heart tissue was cut from the apex. Minced tissue was then digested with collagenase and protease in a 37 ℃ shaker for 7 minutes. The digestion mix was filtered using a 74-μm strainer and centrifuged at 200 g for 5 minutes at 4 ℃. The cell pellet was resuspended by 100 μl FACS buffer, 50 μl was stained with an antibody mixture for 30 minutes on ice in dark. Then each sample was added with 250 μl FACS buffer, filtered with a 74 μm nylon membrane and analyzed by flow cytometer. |
| Instrument | BD FACS Aria Ⅱ |
| Software | Flowjo |
| Cell population abundance | The tissues samples collected weigh approximately equal (about 20mg) and were digested for the same period of time under the same conditions. The digestion mix was filtered, centrifuged and resuspended in same manner for all samples. The cell population after antibody incubation was analyzed at 10th of the sixth power magnitude. |

| | |
|---|---|
| Gating strategy | The preliminary FSC/SSC gate: as the starting cell population.<br>The second PE/SSC gate: CD45 positive was defined as leukocytes.<br>The third APC-Cy7/FITC gate: CD45, CD11b and Ly6G positive were defined as neutrophils. |

☒ Tick this box to confirm that a figure exemplifying the gating strategy is provided in the Supplementary Information.

This checklist template is licensed under a Creative Commons Attribution 4.0 International License, which permits use, sharing, adaptation, distribution and reproduction in any medium or format, as long as you give appropriate credit to the original author(s) and the source, provide a link to the Creative Commons license, and indicate if changes were made. The images or other third party material in this article are included in the article's Creative Commons license, unless indicated otherwise in a credit line to the material. If material is not included in the article's Creative Commons license and your intended use is not permitted by statutory regulation or exceeds the permitted use, you will need to obtain permission directly from the copyright holder. To view a copy of this license, visit http://creativecommons.org/licenses/by/4.0/
